# Community health assets and refugee wellbeing: Qualitative evidence across mental health, disability inclusion, end-of-life care, and women’s health – A global scoping review

**DOI:** 10.1371/journal.pgph.0005459

**Published:** 2026-02-20

**Authors:** Sileshi Demelash Sasie, Melkamu Asrat Alava, Lensa Fekadu, Hailemichael Wasye Misganaw, Tigist Ali Gebeyehu, Neima Zeynu Ali, Sisay Temesgen Dema, Melkamu Abte Afele, Zenebech Mamo Argaw

**Affiliations:** 1 Ethiopian Public Health Institute, Addis Ababa, Ethiopia; 2 Wollo University, Department of Public Health, College of Medicine and Health Sciences, Dessie, Ethiopia; Lebanese American University, LEBANON

## Abstract

Refugee populations face heightened and intersecting vulnerabilities across mental health, disability inclusion, women’s health, and end-of-life and palliative care, shaped by displacement, trauma, legal precarity, and systemic marginalization. Although community-centered approaches are increasingly recognized as critical for addressing these challenges, qualitative evidence on how community health assets support refugee wellbeing remains fragmented across domains. This scoping review aimed to map and synthesize qualitative evidence on community-based health interventions for refugee populations across four thematic areas: mental health, disability inclusion, women’s health, and end-of-life and palliative care. The review followed Joanna Briggs Institute methodology and was conducted in accordance with PRISMA-ScR guidance. A comprehensive search of PubMed, Scopus, and Web of Science identified qualitative studies published between 2010 and 2025. Studies were screened using predefined eligibility criteria, with data charted and thematically analyzed using a cross-domain Consolidated Framework for Implementation Research–Proctor hybrid framework to examine implementation barriers, facilitators, and outcomes. Study quality was appraised using the Critical Appraisal Skills Programme qualitative checklist. The search yielded 2,576 records, of which 1,918 unique records were screened and 126 articles underwent full-text review. Forty-nine studies met inclusion criteria across the four domains. Common barriers included stigma, gender-based violence, legal insecurity, cultural and language discordance, and limited access to specialized services. Effective strategies included peer-led psychosocial support, culturally concordant providers, integrated community-based care models, and safe spaces for women and marginalized groups. Methodological limitations were noted in ethical reporting, researcher reflexivity, and analytic transparency. Across domains, trust, continuity of care, and culturally safe engagement consistently emerged as central to acceptability and feasibility. This review synthesizes cross-domain qualitative evidence and highlights community health assets as critical, scalable platforms for advancing equitable and context-responsive refugee health interventions in both emergency and resettlement settings.

## 1. Background

Globally, millions of forcibly displaced individuals are exposed to profound and largely preventable health risks [1,2]. Refugees, asylum seekers, and internally displaced persons (IDPs) represent legally distinct groups, yet they share a common reality of disrupted lives and fragile health. Refugees are individuals who have crossed an international border because of a well-founded fear of persecution and have been granted protection under the 1951 Refugee Convention and related instruments [3,4]. Asylum seekers are people who have sought this protection but whose claims remain pending, often leaving them in prolonged legal uncertainty and with limited access to essential services [3,5,6]. Internally displaced persons, by contrast, flee similar threats of conflict, violence, or disaster but remain within their country’s borders, where protection and assistance depend largely on domestic systems and the non-binding Guiding Principles on Internal Displacement [4,7,8].

Behind these legal categories are millions of individuals facing profound and preventable health risks. Across settings, forcibly displaced populations experience higher burdens of physical illness, psychological distress, and unmet healthcare needs than surrounding host communities [5–9]. IDPs, in particular, often endure even worse outcomes than refugees, reflecting sudden loss of livelihoods, separation from family networks, and severely constrained access to services [5]. Common barriers to care include administrative exclusion, fear of deportation, lack of documentation, language and cultural obstacles, discrimination, and unaffordable costs [9–12]. These challenges restrict access to primary care, mental health services, sexual and reproductive healthcare, and preventive programs, and are further intensified by overcrowded living conditions, poor water and sanitation, and insecure work [9–11,13].

The scale of this crisis is unprecedented. By 2024, more than 110 million people worldwide were forcibly displaced, with the majority hosted in low- and middle-income countries (LMICs) whose health systems are often overstretched and under-resourced [14,15]. Women and girls nearly half of the global refugee population face additional layers of vulnerability, including gender-based violence, limited reproductive health services, and intersecting social and economic inequalities [16–18]. Across all groups, experiences of violence, loss, and uncertainty accumulate over time, contributing to high levels of depression, anxiety, post-traumatic stress disorder, and other mental health conditions [3–5,13,19]. Recent global shocks, such as the COVID-19 pandemic, have further deepened these inequities by disrupting services, eroding livelihoods, and widening information gaps among displaced communities [10,20].

In response to these challenges, important initiatives have emerged. Asset-Based Community Development (ABCD) approaches in countries such as the United Kingdom and Australia have demonstrated the value of building on community strengths, social networks, and local leadership to support refugee wellbeing [21–23]. Culturally adapted mental health programs, early psychosocial screening, inclusive maternal health services, and digital health tools have shown promise in diverse contexts [14,24,25]. Participatory models such as co-designed services, peer-led networks, and community needs assessments are increasingly recognized as essential for ensuring that interventions are relevant, trusted, and sustainable [23,26,27]. Intersectoral frameworks, including the National Health Service (NHS) Inclusion Health model, illustrate how coordinated action across health, social services, and community organizations can help reduce structural barriers to care [22,28].

Yet progress remains fragmented and uneven. Much of the available research and program experience is concentrated in high-income countries, limiting its applicability to LMICs where most displaced people live [23,29,30]. Studies frequently address single issues in isolation, with little attention to the ways mental health, disability, gender, and age intersect in real lives. Groups with some of the greatest needs people with disabilities, older adults, those requiring palliative care, and adolescent girls are often underrepresented [29–32]. Important areas such as maternal mental health, disability-inclusive services, and end-of-life care remain insufficiently explored. Moreover, many interventions lack grounding in implementation science frameworks and rarely assess long-term outcomes or system-level enablers [33]. A persistent absence of meaningful refugee participation in research and program design further limits cultural appropriateness and practical impact [9,34–36].

Qualitative evidence is uniquely positioned to address these shortcomings. By capturing lived experiences, community perspectives, and the everyday realities of service delivery, qualitative studies illuminate dimensions of access, trust, and dignity that quantitative indicators alone cannot reveal [37,38]. However, this evidence remains scattered across separate thematic silos, with few efforts to synthesize insights across domains.

This scoping review seeks to close that gap. For the first time, it brings together qualitative evidence across four critical areas of refugee health mental health, disability inclusion, women’s health, and end-of-life and palliative care. By integrating findings from diverse settings and perspectives, the review identifies shared barriers, practical facilitators, and cross-cutting lessons for implementation. In alignment with World Health Organization (WHO) and United Nations High Commissioner for Refugees (UNHCR) priorities for equity-oriented and culturally responsive care [39], the review aims to generate practical and actionable insights to inform inclusive, participatory, and context-sensitive interventions for displaced populations worldwide.

## 2. Methods

### 2.1 Ethics statement

This study is a scoping review of peer-reviewed, publicly available literature and did not involve the collection of primary data or direct engagement with human participants. In accordance with international ethical guidelines for secondary research, institutional ethical approval and informed consent were not required.

### 2.2 Study design and conceptual framework

This review focused exclusively on qualitative studies to capture context-sensitive evidence on refugee experiences, perceptions, and priorities that are not readily addressed through quantitative designs [37,40]. Qualitative research is particularly valuable for understanding the cultural, social, and systemic factors that shape access to care, service acceptability, and implementation of health interventions in complex displacement settings.

The review was conducted in accordance with the Joanna Briggs Institute (JBI) methodology for scoping reviews, which provides a structured and transparent approach for mapping the breadth and depth of existing research [41]. Methodological guidance from Arksey and O’Malley [42] and Levac et al. [43] was applied to strengthen rigor in study selection, data extraction, and synthesis processes. Reporting of the review followed the PRISMA-ScR (Preferred Reporting Items for Systematic Reviews and Meta-Analyses extension for Scoping Reviews) guideline [44].

The synthesis was further informed by established implementation science frameworks. The Consolidated Framework for Implementation Research (CFIR) and Proctor’s implementation outcomes were used to guide interpretation of findings and to identify determinants of intervention feasibility, acceptability, and sustainability [29,37,38].

### 2.3 Objectives and research questions

The objective of this scoping review was to systematically map and synthesize qualitative evidence on implementation experiences, challenges, and outcomes of health interventions for refugee and forcibly displaced populations across four priority domains: mental health, disability inclusion, women’s health, and end-of-life/palliative care.

The CFIR and Proctor’s Implementation Outcomes Framework guided the identification of implementation determinants, strategies, and reported outcomes. Research questions were developed in alignment with the Population–Concept–Context (PCC) framework, with the *Population (P)* fixed as refugee and forcibly displaced populations. The *Concept* and *Context* elements varied across questions, as shown in **Table 1**.

**Table 1:** Research Questions Aligned with the PCC Framework.

Research Question	PCC Element(s)
1. What barriers and facilitators influence the implementation of health interventions targeting refugee populations in the four focal domains?	Concept, Context
2. What are the core characteristics of effective or innovative interventions across mental health, disability inclusion, women’s health, and palliative care settings?	Concept
3. How are participatory approaches (e.g., co-design, community engagement) integrated into intervention delivery for refugee populations?	Concept, Context
4. In what ways do cross-sectoral collaborations (e.g., health, protection, education) support or hinder implementation success?	Context
5. What outcomes have been reported in terms of acceptability, feasibility, reach, and sustainability of these interventions?	Concept
6. Where are the persistent evidence gaps in implementation-focused qualitative research on refugee health?	Concept, Context

### 2.4 Eligibility criteria

The scope of eligible studies was structured using the Population–Concept–Context (PCC) framework recommended for scoping reviews. This framework guided the formulation of inclusion and exclusion criteria and ensured alignment between the review objectives and study selection processes. The PCC parameters defining the scope of this review are summarized in **Table 2**.

**Table 2:** PCC Framework Defining the Scope of the Scoping Review.

Element	Description	Application to This Review
**Population**	Individuals and groups forcibly displaced due to conflict, persecution, or disasters.	Refugees, asylum seekers, and internally displaced persons (IDPs), regardless of age, gender, or country of origin.
**Concept**	Community health assets and wellbeing across four priority health domains.	Focus on qualitative evidence addressing: Mental health Disability inclusion End-of-life and palliative care Women’s health Also includes participatory models, asset-based approaches, and intersectional subgroup analysis.
**Context**	Global, with emphasis on disparities between low-, middle-, and high-income countries.	Health systems, community settings, resettlement environments, and humanitarian contexts in both resource-rich and resource-limited settings.

#### Inclusion criteria.

- **Study design:** Qualitative studies (e.g., interviews, focus groups, ethnography, phenomenology, participatory co-design) reporting primary data.
- **Population:** Human participants who are refugees, asylum seekers, or internally displaced persons (IDPs).
- **Focus:** Addresses at least one of the four focal domains mental health, disability inclusion, end-of-life/palliative care, or women’s health.
- **Child and adolescent populations:** Studies involving children and adolescents were eligible when they addressed issues directly relevant to one of the four focal domains (for example, mental health of refugee youth or disability-related services for children). Broader pediatric health topics not linked to these domains were considered outside the scope of this review.
- **Publication type:**
  ◦ Peer-reviewed journal articles published between January 2010 and December 2025, in English.
  ◦ Grey literature outputs (e.g., reports, policy documents, program evaluations, and guidelines from international organizations, NGOs, and government bodies) that present primary qualitative data.
- **Accessibility:** Full-text documents available at the time of screening

#### Exclusion criteria.

- Quantitative or mixed-methods studies without disaggregated qualitative findings.
- Studies not centered on forcibly displaced populations.
- Grey literature without methodological transparency (e.g., blogs, opinion pieces, media reports) and non-peer-reviewed conference abstracts.

### 2.5 Information sources and search strategy

A comprehensive search strategy was developed to identify both peer-reviewed and grey literature relevant to the review objectives. For the peer-reviewed component, three bibliographic databases PubMed, Web of Science, and Scopus were systematically searched. Search strategies combined controlled vocabulary (e.g., MeSH in PubMed) with free-text terms reflecting four core concepts: displaced populations, focal health domains, qualitative methods, and implementation. Boolean operators (AND/OR) were applied, and search fields were adapted for each database, including TITLE-ABS-KEY in Scopus and TS = Topic in Web of Science.

To complement the academic search, targeted strategies were applied to global repositories and organizational portals in order to capture relevant grey literature. Sources included the WHO Institutional Repository for Information Sharing (IRIS), the World Bank Documents Portal, UNHCR publications, and International Organization for Migration (IOM) resources. Focused Google site searches were also conducted for humanitarian organizations such as United Nations Children’s Fund (UNICEF), Médecins Sans Frontières (MSF), and Save the Children. These searches incorporated refinements such as document-type keywords (e.g., report, framework, guideline, policy) and file-type filters (filetype:pdf) to maximize relevance and precision.

Additional measures were undertaken to ensure completeness and reduce the risk of missing eligible studies. Google Scholar was searched systematically using the same conceptual terms, and records were screened sequentially until thematic saturation was reached, which corresponded to approximately the first 200 results. Citation tracking was also employed: backward citation tracking involved screening the reference lists of included studies, while forward citation tracking used the Google Scholar “Cited by” function to identify more recent relevant works. The complete search strategies for all databases and repositories, including Boolean strings, refinements, and applied filters, are provided in S1 Table.

### 2.6 Study selection

All identified records were imported into EndNote, where duplicates were removed prior to screening. The selection process was carried out in two phases: an initial title and abstract screening followed by full-text review. Two reviewers (SDS and FMA) independently assessed all records against the predefined eligibility criteria. To ensure consistency, a calibration exercise was conducted on a random sample of ten records at the title and abstract stage. Reviewers agreed on nine of the ten records (90% raw agreement), and Cohen’s kappa [45] was calculated at 0.82, indicating substantial agreement according to the benchmarks established by Landis and Koch [46].

**Fig 1:**
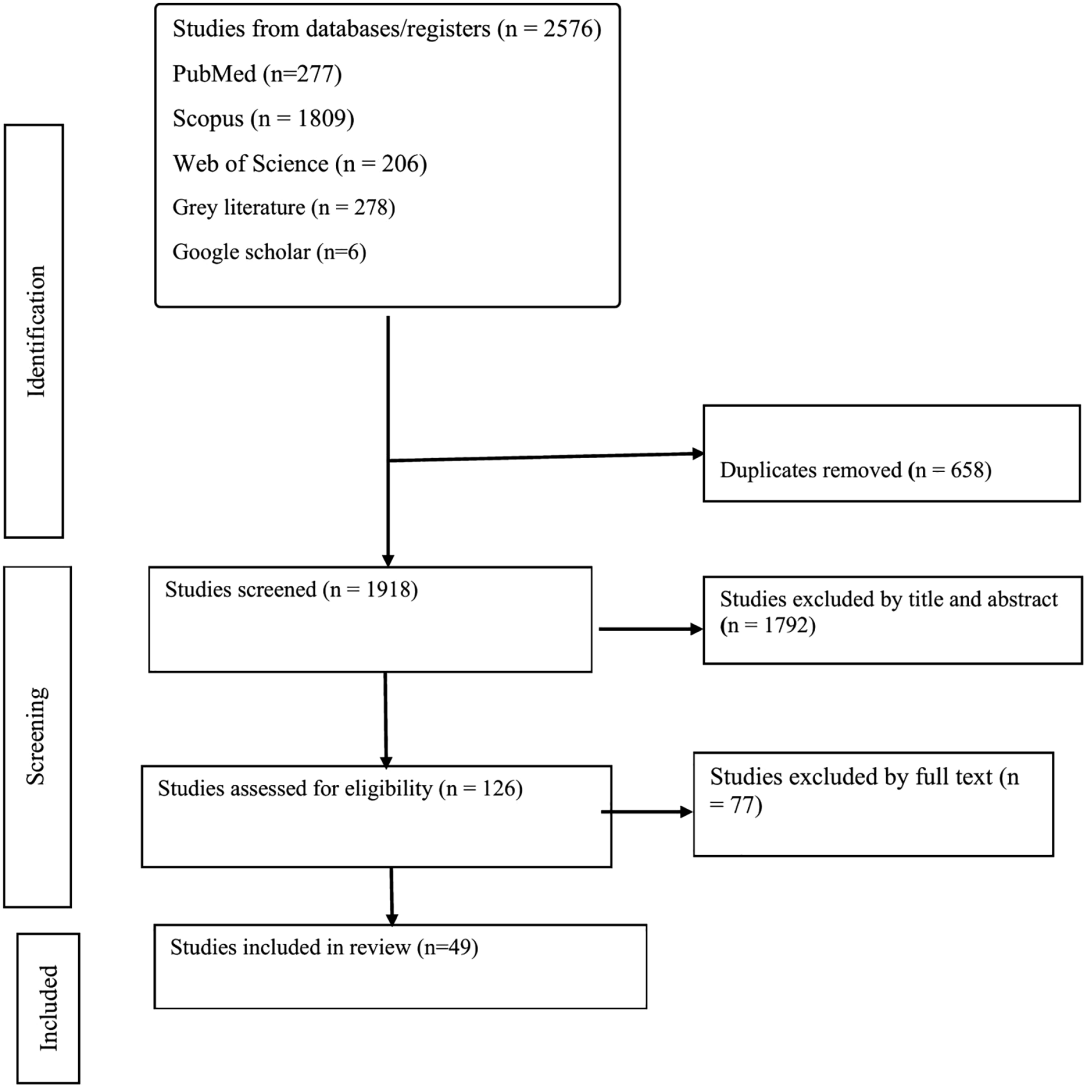
PRISMA flow diagram of the study selection process.

Following calibration, the full screening process proceeded independently. Any discrepancies between reviewers were resolved through discussion, and unresolved cases were adjudicated by a third reviewer (HWM). The selection process, including reasons for exclusion at each stage, is detailed in the PRISMA-ScR flow diagram (Fig 1**).**

### 2.7 Data extraction

Data extraction was conducted using a structured and pre-defined framework developed specifically for this scoping review. The framework was designed to capture key methodological characteristics, contextual information, and implementation-relevant evidence in a consistent and reproducible manner across all eligible studies.

For each included study, data were systematically extracted using the following fields: reference (authors and year of publication), country or setting, thematic domain, primary population, age group, gender composition, pertinent findings, study-reported limitations, and authors’ recommendations. These variables were selected to support transparent description of study characteristics and to enable consistent synthesis across the four thematic domains of the review.

Primary population was coded according to the target group described in each study, including refugees, asylum seekers, internally displaced persons, or mixed populations. Age was categorized based on the descriptors used by study authors (e.g., children, adolescents, adults, older adults, or mixed-age groups). Gender composition was recorded as women-only, men-only, mixed-gender, or not reported. Geographic setting was captured at the country or regional level as specified in the original publication.

Data extraction was performed independently by two reviewers (SDS and FMA) using a standardized extraction template. Discrepancies between reviewers were resolved through discussion, and when consensus could not be reached, a third reviewer (HWM) adjudicated the final decision. All extracted data were entered into a centralized database to ensure uniformity and to support subsequent synthesis.

For each included study, data were systematically extracted using a standardized set of variables designed to capture study characteristics and relevant qualitative information. The complete list of extraction variables is provided in S2 Table.

### 2.8 Data synthesis

Data synthesis was conducted using a structured thematic synthesis approach consistent with the method described by Thomas and Harden [47]. The process combined inductive coding of qualitative data with deductive organization using established implementation science frameworks. All information extracted in the “pertinent findings” field was first reviewed in its original qualitative form and coded inductively to capture key concepts, processes, and contextual factors described in each study.

Following inductive coding, the resulting codes were organized deductively using the Consolidated Framework for Implementation Research (CFIR) domains [48] to classify implementation determinants and Proctor’s implementation outcomes [49] taxonomy to categorize reported outcomes. This dual-framework approach provided a consistent analytic structure for organizing evidence on intervention characteristics, contextual influences, and implementation processes across diverse study settings.

Synthesis was undertaken in two sequential stages. In the first stage, coded data were organized and analyzed within each predefined thematic domain to generate domain-specific themes. In the second stage, a cross-domain synthesis was conducted to integrate evidence across domains and to identify commonalities and differences in implementation experiences.

Studies addressing more than one thematic domain were coded under all applicable domains during data extraction. For within-domain analyses, findings from these studies were included in each relevant domain. However, during cross-domain synthesis and any aggregate summaries, each study was treated as a single analytic unit and counted only once, regardless of the number of domains to which it contributed.

Demographic variables specified for inclusion in the synthesis were age group, gender composition, and geographic setting. Procedures were established to record these variables exactly as reported in the primary studies, using the categories and descriptors provided by the original authors. Where any demographic information was absent from a publication, it was to be coded as “not reported” rather than inferred. These rules were applied consistently to maintain methodological transparency.

Synthesized data were organized using narrative summaries and structured analytic matrices to enable systematic comparison across studies and thematic domains. These matrices were used to collate coded information, map implementation determinants and outcomes, and facilitate consistent integration of qualitative evidence.

### 2.9 Quality appraisal

To assess methodological rigor, all included studies were appraised using the Critical Appraisal Skills Programme (CASP) Qualitative Studies Checklist [50]. This checklist evaluates ten domains, including clarity of the research aim, appropriateness of qualitative methods, justification of research design, recruitment strategies, alignment between data collection and study objectives, researcher reflexivity, ethical considerations, rigor of data analysis, clarity of findings, and overall research value.

Although formal quality assessment is not a mandatory component of scoping reviews, the inclusion of CASP provided a structured appraisal of methodological strengths and limitations across studies. In accordance with established scoping review methodology, quality appraisal was conducted to describe the methodological characteristics of the evidence base rather than to exclude studies on the basis of quality [47,51].

## 3. Results

### 3.1 Study selection summary

The study selection process is presented in Fig 1 (PRISMA-ScR flow diagram). The initial database search identified 277 records from PubMed, 1,809 from Scopus, and 206 from Web of Science, yielding a total of 2,292 records. An additional 278 records were retrieved from grey literature sources and 6 from Google Scholar, resulting in 2,576 records overall. After removal of 658 duplicates, 1,918 unique records remained for title and abstract screening. Of these, 1,793 were excluded, leaving 126 articles for full-text review. Following detailed assessment, 77 articles were excluded, resulting in 49 studies that met the inclusion criteria and were retained for the final scoping review (Fig 1).

### 3.2 Study characteristics

The 49 included studies provided a broad but unevenly distributed body of qualitative evidence across the four domains of mental health, disability inclusion, women’s health, and end-of-life and palliative care. Collectively, the studies spanned diverse contexts, including high-income resettlement countries, low-resource humanitarian settings, and transnational migration environments. Geographically, Europe (n = 14) and North America (n = 13) contributed the largest share of studies, with representation across all four domains. Africa (n = 6) and the Middle East (n = 6) offered moderate coverage, each encompassing at least three domains. Oceania (n = 6) was represented primarily in women’s health and end-of-life research, with no studies addressing disability inclusion. Asia (n = 3) contributed exclusively to the end-of-life domain, while South America (n = 1) was represented by a single study focused on disability inclusion.

Study population characteristics varied across the included literature. Demographic reporting was inconsistent, with most studies focusing on adult populations and a smaller subset addressing children, youth, or older adults. Thirteen studies examined women-only samples, particularly within the women’s health and gender-sensitive mental health domains, while others included mixed-gender populations or did not clearly specify gender composition. The evidence base reflects diverse geographic regions, including Europe, North America, Africa, the Middle East, Asia, and Australia.

Mental health studies (n = 12) frequently examined culturally adapted psychosocial interventions, stigma reduction strategies, faith-based supports, and digital tools intended to improve service accessibility and acceptability. Disability inclusion research (n = 11) often focused on enhancing accessibility for refugees with disabilities, with particular emphasis on inclusive program design, early developmental screening, and intersectional approaches addressing both gender- and disability-related needs. Women’s health studies (n = 9) highlighted culturally tailored maternal and perinatal care, peer-led health promotion initiatives, faith-integrated mental health interventions, and community-led service models aimed at improving engagement and trust in healthcare systems. End-of-life and palliative care studies (n = 17) explored dignity-preserving models of care, the role of family and community engagement, integration with primary healthcare, and context-specific approaches to service delivery in both camp-based and urban host settings.

Across all four domains, studies reported recurring patterns related to cultural competence, community engagement, and participatory approaches. Frequently reported constraints included language and communication challenges, stigma, discriminatory practices, restrictive policy environments, and fragmented service delivery systems. Many studies also documented practical recommendations to address these challenges, including expansion of peer support networks, cross-sectoral collaboration, culturally and linguistically appropriate provider training, and alignment of services with the social and cultural contexts of refugee populations. A detailed synthesis of individual study characteristics, demographic information, key findings, limitations, and recommendations is presented in S3 Table.

### 3.3 Quality appraisal of included studies

All 49 qualitative studies included in the review were appraised using the CASP Qualitative Studies Checklist, in line with scoping review methodology, which emphasizes mapping the evidence base rather than excluding studies on the basis of quality. Overall, most studies demonstrated clearly articulated aims, appropriate use of qualitative methodology, and data collection methods that were consistent with their objectives.

However, variability was observed across several critical domains. Recruitment strategies were frequently insufficiently described [52–54], limiting transparency around sampling processes and the potential for bias. Ethical considerations were also underreported in many cases; several studies failed to specify informed consent procedures or safeguards for vulnerable populations, particularly displaced women, persons with disabilities, and survivors of trauma [55,56].

A recurring limitation across the dataset was the lack of reflexivity. More than half of the studies provided minimal or no discussion of researcher positionality or the dynamics between researchers and participants, despite the importance of such considerations in refugee health research [57–59]. In addition, analytic rigor varied considerably. Some older and even more recent studies [60,61] presented only surface-level descriptions of data analysis, with limited detail on coding strategies, triangulation, or validation processes.

Nevertheless, the majority of studies offered clear statements of findings and contributed valuable insights, with several [58,62–64] demonstrating consistently high methodological quality across appraisal domains. Collectively, these results highlight both the strengths of the existing evidence and the key areas requiring improvement in future qualitative research particularly enhanced transparency in ethical reporting, stronger reflexivity, and more robust descriptions of analytic processes. A summary of quality appraisal findings is provided in S4 Table.

### 3.4 Domain-level findings: refugee health interventions across four contexts

Evidence on refugee health interventions clustered around four prominent domains: mental health, disability inclusion, women’s health, and end-of-life/palliative care. These domains capture intersecting determinants of health and align with global priorities for displaced populations. Across domains, mapping interventions onto CFIR constructs and Proctor’s outcomes revealed consistent challenges, including structural exclusion within the Outer Setting, organizational and infrastructural gaps within the Inner Setting, and the persistent need for participatory processes to ensure that interventions are contextually adapted to refugee realities. The synthesis below presents domain-specific findings while identifying recurrent patterns across interventions that directly address the guiding questions of this review.

#### 3.4.1 Mental health.

Twelve studies examined refugee mental health interventions, with most focusing on psychosocial support, trauma recovery, stigma reduction, and community-based care [52–55,65–71]. Intervention models encompassed culturally congruent psychosocial programs, faith-integrated supports, stigma-sensitive digital tools, and trauma-informed approaches [53–55,66]. Collectively, the evidence highlights that intervention effectiveness was contingent on the alignment between program design and the broader socio-political and organizational contexts in which refugees were situated.

Across CFIR domains, the Outer Setting exerted the strongest influence. Legal precarity, asylum process stress, unstable housing, and stigma systematically undermined service continuity [52,57]. These constraints help explain why even well-adapted psychosocial interventions often struggled in the absence of broader protections. At the Inner Setting level, dependence on NGO-based services, weak referral networks, and under-resourced health facilities limited integration into host-country systems [65,70]. Programs that incorporated cultural competency training for general practitioners and frontline providers reported greater feasibility and adoption [70,71], underscoring how organizational readiness interacts with provider competence to shape implementation outcomes.

Characteristics of individuals were also critical. Distrust, shame, and cultural taboos around mental illness discouraged service uptake [53–55], while resilience and faith-based coping facilitated engagement [54,68]. Provider cultural competence emerged as a key mediator: interventions faltered where providers lacked cross-cultural awareness but flourished when training and collaboration helped bridge cultural divides [70].

Process strategies such as participatory co-design, peer-led delivery, and community involvement consistently enhanced intervention acceptability and contextual fit [67,71]. Digital interventions revealed a nuanced pattern: stigma-aware, human-centered approaches increased reach and engagement [55], but digital divides stemming from limited internet access, literacy barriers, and trust concerns restricted feasibility in many settings.

When mapped to Proctor’s outcomes, interventions were consistently rated acceptable and appropriate when culturally and linguistically tailored [66,68]. Feasibility and adoption improved when programs employed community facilitators and culturally competent staff [67,70], while fidelity was reinforced by embedding cultural sensitivity into implementation protocols. However, penetration and sustainability were rarely achieved, as these outcomes were contingent on integration with broader supports such as housing, legal aid, and policy reforms [57,65].

Reported outcomes included reductions in psychological distress and trauma symptoms [66], strengthened coping and resilience [54,68], improved help-seeking and social connectedness [52,67], enhanced cultural competence among providers [70,71], and symptom reduction through tailored digital programs [55]. Despite these promising findings, few studies evaluated long-term continuity, highlighting a critical evidence gap regarding sustainability and systemic integration (**Table 3**).

**Table 3:** Summary of qualitative evidence on mental health interventions for refugee populations mapped to CFIR constructs, Proctor’s implementation outcomes, barriers, facilitators, and reported outcomes.

Author(s)	Context	CFIR Summary	Proctor Summary	Barriers	Facilitators	Outcomes
Robinson et al. (2021) [53]	Rwanda & Uganda; Congolese refugees in camps	Outer: camp life constraints; Intervention: stigma reduction linked to personal appearance; Individuals: shame impacting mental health engagement	High acceptability and appropriateness when dignity and personal identity are addressed	Structural inequities and limited access to resources hinder intervention delivery	Strong community cohesion and culturally relevant approaches enhance engagement	Increased self-worth and improved social relationships
Kienzler (2024) [65]	London, UK; refugee/asylum MHPSS providers	Outer: hostile asylum policy environment; Intervention: integrated service delivery; Inner: NGO-based provision; Process: participatory co-design	Appropriateness constrained by restrictive policies; sustainability requires systemic reform	Hostile policy environment and inequitable asylum processes	Rights-based frameworks and participatory service design improve acceptability	Better service integration if policy barriers are removed
Paudyal et al. (2021) [66]	UK; Syrian adult refugees	Outer: stigma and language barriers; Intervention: culturally congruent psychosocial support; Individuals: reliance on faith and self-agency	Culturally congruent approaches acceptable; community-based delivery feasible	Language barriers, stigma, and low awareness of available services	Faith practices, nature-based coping, and community solidarity	Strengthened social connectedness and emotional relief
Walther et al. (2021) [52]	Germany; asylum seekers/refugees	Outer: uncertainty of asylum process and bureaucratic demands; Inner: pressure to integrate rapidly	Appropriateness dependent on addressing integration challenges	Bureaucratic stress and social isolation reduce engagement	Community support networks and individual resilience improve participation	Enhanced mental health outcomes through better integration support
Laham et al. (2020) [67]	North Lebanon; Syrian refugees and Lebanese host community	Outer: stigma and limited-service access; Intervention: accessible, stigma-reducing services; Individuals: culturally influenced help-seeking patterns	Acceptable if stigma addressed; feasible when physically and financially accessible	Stigma and geographical/economic access barriers	Community education and trusted local providers facilitate uptake	Increased help-seeking behaviors
Bridi et al. (2023) [68]	USA & Jordan; aging Arab refugees	Outer: cultural norms shaping perceptions; Intervention: faith-integrated mental health services; Individuals: faith-based coping	Faith-integrated approaches are highly acceptable	Cognitive stigma and fear of dementia reduce uptake	Faith, scripture, and spiritual resilience enhance participation	Improved coping and illness understanding
Khan et al. (2022) [57]	Canada; homeless refugee youth	Outer: unstable housing and immigration status; Inner: high trauma exposure; Individuals: stress resilience	Appropriateness relies on securing stable housing	Housing insecurity and social isolation impede continuity	Youth-focused supports and mentorship encourage sustained engagement	Increased resilience and coping capacity
Ahmed Z et al. (2024) [69]	Kenya; Somali refugees in urban areas	Outer: war trauma and chronic poverty; Intervention: trauma-informed community mental health; Individuals: high community stress	Trauma-informed interventions are acceptable	Chronic adversity and fragmented service infrastructure limit implementation	Stakeholder engagement and peer support promote acceptability	Foundations laid for tailored future interventions
Jensen et al. (2013) [70]	Denmark; GPs treating refugees	Intervention: cultural competency training for primary care providers; Inner: limited readiness; Individuals: awareness and skill gaps	Cultural competency training essential for acceptability and feasibility	Lack of GP preparedness and communication barriers	Targeted training and inter-professional collaboration	Improved care delivery for refugees
Silver et al. (2023) [71]	Wales, UK; refugees & medical students	Outer: training gaps in refugee health; Intervention: direct refugee involvement; Process: experiential learning	High acceptability and appropriateness with refugee involvement in training	Lack of experiential learning opportunities	Direct refugee engagement in training design	Greater empathy and cultural awareness among trainees
Fabian et al. (2023) [55]	USA; immigrant/refugee youth	Intervention: tailored, stigma-aware digital mental health; Process: human-centered co-design	High acceptability, adoption, and feasibility	Limited internet access and trust in digital tools	Culturally adapted content and personalization	Increased engagement and symptom reduction
Shannon et al. (2015) [59]	USA; multiple refugee groups	Outer: histories of repression; Intervention: stigma-reduction programming; Individuals: shame and distrust of providers	Low acceptability unless stigma is directly addressed	Deep-seated shame, mistrust, and avoidance	Education and peer-led stigma reduction	Increased recognition and discussion of stigma barriers

#### 3.4.2 Disability inclusion.

Eleven studies explored disability-inclusive interventions for refugees with physical, sensory, or cognitive impairments, often intersecting with age, gender, and trauma histories. Interventions included accessibility audits, inclusive health outreach, community-based rehabilitation, and peer-led advocacy. While these programs addressed multiple CFIR domains and Proctor outcomes, the synthesis shows that most operated as corrective measures within systems marked by structural neglect, highlighting persistent gaps in disability recognition, resource allocation, and policy integration.

Outer Setting determinants were particularly pronounced. Policy gaps, unsafe migration conditions, entrenched stigma, and systemic neglect of disability needs consistently undermined implementation [56,72,73]. These external barriers disproportionately affected refugee women with disabilities, who frequently faced intersectional discrimination [56]. Nonetheless, promising adaptations emerged: coordinated disability service models along migration routes [73] and tailored -prevention programs for persons with communication disabilities [74] demonstrated how context-specific approaches, when aligned with humanitarian or policy frameworks, could mitigate risks.

At the Inner Setting level, constraints reflected inadequate provider training, inaccessible infrastructure, and weak intersectoral coordination [62,75]. Interventions struggled where organizations lacked adaptive facilities or case management systems. By contrast, inclusive classroom models in Germany illustrated how organizational culture, teacher preparedness, and peer inclusion could significantly enhance adoption [63]. These variations underscore that organizational readiness is not merely a supportive factor but a determinant of feasibility and sustainability.

Individual characteristics and process strategies also shaped outcomes. Provider competence, cultural sensitivity, and advocacy skills were critical for engagement, while refugees’ resilience and agency contributed to uptake [76,77]. Programs incorporating participatory design, caregiver engagement, and partnerships with disability organizations achieved stronger contextual fit and legitimacy [63,77]. Peer networks and leadership initiatives particularly those led by women with disabilities were transformative, enhancing both acceptability and social participation [72].

Mapped to Proctor’s outcomes, interventions demonstrated high appropriateness when grounded in inclusive communication and intersectional design [74,77]. Acceptability improved through peer support and culturally adapted screening [76]. Feasibility was frequently undermined by inaccessible facilities and fragmented services [62,75], though community navigation and teacher training enhanced adoption [63,76]. Fidelity was best maintained when inclusion principles were embedded into program protocols, while penetration and sustainability remained limited, dependent on systemic integration and stable funding. Reported impacts included earlier developmental screening [76], improved caregiver coping [78], increased reporting of gender-based-violence (GBV) [74], and enhanced educational and social participation [63,72].

Barriers were consistently systemic and multi-level, including inaccessible infrastructure, lack of assistive devices, transportation challenges, siloed disability services, migration hazards, and institutional gatekeeping [62,73,75]. Facilitators included interpreter provision, inclusive communication, awareness campaigns, and advocacy-led program design [74,77]. Cross-sector collaboration particularly between humanitarian and medical actors and empowerment initiatives further supported uptake and sustainability [63,72].

Taken together, these findings suggest that while disability-inclusive interventions generate meaningful gains across health, education, and protection sectors, they remain structurally fragile. Success depended less on the specific features of individual programs than on their ability to challenge systemic neglect and foster sustained cross-sector collaboration. The evidence underscores a persistent gap between short-term adaptation and long-term institutional reform, pointing to the need for systemic accountability and durable inclusion mechanisms within both humanitarian and host-country systems (**Table 4**).

**Table 4:** Summary of qualitative evidence on disability inclusion interventions for refugee populations mapped to CFIR constructs, Proctor’s implementation outcomes, barriers, facilitators, and reported outcomes.

Citation	Context	CFIR Summary	Proctor Summary	Barriers	Facilitators	Outcomes
Marshall & Barrett (2025) [74]	Rwanda; refugees with communication disabilities; prevention/response to sexual and gender-based violence	Outer: risks, policy gaps, harmful norms; Intervention: tailored prevention adaptable to disabilities; Inner: organizational infrastructure for inclusion; Individuals: skills, motivation, cultural competence; Process: outreach and community engagement	High appropriateness when accessible communication is used	Communication barriers, stigma	Inclusive communication methods, community awareness campaigns	Improved reporting rates and prevention awareness
Mirza et al. (2013) [79]	US Midwest; refugees with disabilities and chronic health conditions	Outer: healthcare access barriers, policy gaps; Intervention: inclusive service design; Inner: coordination gaps; Individuals: staff/client awareness; Process: planning and evaluation	Low feasibility without adaptation	Transportation, cost, inaccessible facilities	Community-based health navigation support	Identified service gaps, integration recommendations
Kroening et al. (2016) [76]	USA; developmental screening for refugee children	Outer: need for early identification; Intervention: screening protocols; Inner: clinic readiness; Individuals: cultural competence of providers; Process: implementation and evaluation	High adoption in clinics with trained staff	Language barriers, cultural unfamiliarity	Interpreter support, culturally adapted tools	Earlier identification of developmental needs
Tofani et al. (2025) [73]	Italy; refugees with disabilities along migration routes	Outer: migration hazards, policy gaps; Intervention: disability-inclusive migration services; Inner: shelter service gaps; Individuals: staff/volunteer sensitivity; Process: planning and engagement	Appropriateness improved by coordinated disability services	Unsafe routes, lack of medical continuity	Humanitarian-medical collaboration	Policy recommendations for disability-inclusive migration
Harris & Roberts (2003) [77]	Multiple settings; disabled refugees in research participation	Outer: ethical and inclusion barriers; Intervention: inclusive research/program design; Inner: infrastructure readiness; Individuals: advocacy skills; Process: participatory design	Acceptability improved by inclusive recruitment	Physical inaccessibility, gatekeeping	Accessible venues, advocacy involvement	Increased participation in research
Avci & Sengul (2024) [56]	Turkey; Syrian refugee women with disabilities	Outer: gendered disability stigma; Intervention: gender- and disability-responsive programs; Inner: infrastructure readiness; Individuals: resilience, skills; Process: community engagement	Appropriateness improved with intersectional approach	Intersectional discrimination	Peer support networks	Highlighted need for gender-disability policy
Mirza & Heinemann (2012) [62]	USA; refugees with disabilities post-resettlement	Outer: fragmented services, coordination gaps; Intervention: integrated service models; Inner: case management gaps; Individuals: provider awareness; Process: planning and delivery	Feasibility hindered by siloed systems	Fragmented services	Integrated case management	Identified priorities for integration
Bacakova (2025) [63]	Germany; Ukrainian refugee children with disabilities in schools	Outer: policy support for inclusive education; Intervention: inclusive classrooms; Inner: supportive school culture; Individuals: teacher competence; Process: training and peer inclusion	High adoption in inclusive schools	Language, cultural barriers	Teacher training, peer inclusion	Improved learning and integration
Fayad et al. (2024) [78]	Jordan; refugee caregivers using mindfulness with disabled children	Outer: caregiver stress, limited support; Intervention: mindfulness program; Inner: organisational support for mental health; Individuals: caregiver openness; Process: training delivery	High acceptability and feasibility	Limited program reach	Accessible training materials	Improved caregiver coping skills
Serrano & Martin (2021) [75]	Brazil; disabled refugees and healthcare access	Outer: healthcare inequities, policy gaps; Intervention: equity-focused care models; Inner: infrastructure barriers; Individuals: provider advocacy; Process: planning and execution	Feasibility improved by universal design	Policy-execution gap	Civil society advocacy	Policy reform recommendations
Scheer & Mondaca (2022) [72]	Europe; refugee women with disabilities and social participation	Outer: societal discrimination; Intervention: empowerment and leadership programs; Inner: inclusive culture; Individuals: agency, motivation; Process: program delivery	Acceptability enhanced by empowerment programs	Discrimination, exclusion	Leadership programs, empowerment initiatives	Improved participation and confidence

#### 3.4.3 Women’s health.

Nine studies addressed women’s health interventions among refugee populations, focusing on maternal health, reproductive autonomy, perinatal care, and GBV prevention. Intervention designs included culturally tailored maternal health models, integrated antenatal services, peer-led health promotion, and faith-based approaches.

Outer Setting determinants reported across studies included socio-economic hardship, restrictive policies, gendered stigma, and fragmented maternal health services [80,81]. These factors were associated with reduced continuity of care and lower service utilization, particularly among undocumented women and those in transit. Some studies reported that programs linking health services with social supports, such as housing assistance and NGO partnerships, improved access [80].

At the Inner Setting level, organizational capacity and provider preparedness varied. Facilities lacking gender-sensitive infrastructure or staff trained in culturally responsive care were associated with disengagement [81,82]. In contrast, programs employing female providers, peer navigators, and community health workers were reported as demonstrating higher feasibility and adoption [83,84]. Most initiatives were described as pilot projects and were not integrated into national maternal health frameworks.

Individual characteristics and process strategies influenced implementation. Several studies described refugee women’s resilience and leadership as facilitating program uptake, particularly in peer-led and co-leadership models [58,81]. In contrast, mistrust, fear, and digital exclusion limited engagement [58]. Participatory co-design and community leadership were reported as enhancing contextual fit, while digital interventions produced mixed results due to connectivity and literacy challenges [58].

Across Proctor’s outcomes, acceptability and appropriateness were reported as higher when services were linguistically and culturally tailored [82,83]. Feasibility and adoption improved with peer involvement and continuity of care [83,84]. Penetration and sustainability were less frequently reported; stand-alone pilots often ended once external support was withdrawn, whereas integration with policy frameworks and partnerships was associated with greater continuity [80,81].

Overall, the nine studies reported outcomes such as increased service uptake, improved engagement with providers, enhanced trust through peer-led initiatives, and context-specific adaptations to maternal and reproductive health services (**Table 5**).

**Table 5:** Summary of qualitative evidence on women’s health interventions for refugee populations mapped to CFIR constructs, Proctor’s implementation outcomes, barriers, facilitators, and reported outcomes.

Citation	Context	CFIR Summary	Proctor Summary	Barriers	Facilitators	Outcomes
Kasper et al. (2021) [82]	Multiple countries; maternal health care for refugee women	Outer: systemic barriers to maternal care, policy and cultural constraints; Intervention: adaptable, culturally tailored maternal health models; Inner: facility readiness, communication networks, leadership; Individuals: provider competence, patient trust; Process: limited engagement and evaluation	High acceptability and appropriateness when culturally adapted; adoption hindered by systemic gaps	Language barriers, cultural incongruence, cost	Cultural mediators, trained interpreters, women-centered models	Improved maternal care uptake where models adapted
Adıbelli & Şahan (2025) [80]	Turkey; refugee women and children	Outer: socio-economic stressors, housing and integration issues; Intervention: multi-sector health and social support; Inner: coordination gaps; Individuals: resilience, coping strategies; Process: planning, engagement, evaluation	Appropriateness linked to multi-sector integration	Economic hardship, limited healthcare access	Community support, NGO assistance	Increased access to integrated support services
Griffin et al. (2022) [58]	Australia; Myanmar refugee-background women	Outer: information access barriers; Intervention: peer-led, co-designed health information; Inner: social support networks, organizational readiness; Individuals: literacy, trust; Process: community engagement and evaluation	High acceptability and feasibility for peer-led programs	Language barriers, digital divide	Peer educators, culturally tailored materials	Improved health knowledge and service navigation
Babatunde-Sowole et al. (2020) [84]	Australia; health screening and preventative care	Outer: barriers to preventive service uptake; Intervention: culturally adapted screening; Inner: clinic workflow integration; Individuals: cultural awareness, self-efficacy; Process: program delivery and evaluation	Feasibility and adoption improved with cultural adaptation	Fear, mistrust, low awareness	Female providers, culturally sensitive approaches	Higher screening participation and trust
Due et al. (2022) [83]	Australia; perinatal care for African-background refugee women	Outer: gaps in maternal mental health support, policy/funding limits; Intervention: integrated perinatal care; Inner: continuity of care culture; Individuals: trust, cultural norms; Process: participatory input	High acceptability and appropriateness for integrated care	Stigma, cultural mismatch	Continuity of care, trust-building	Improved maternal mental health outcomes
Woodgate et al. (2017) [81]	Canada; African immigrant/refugee families in PHC	Outer: access barriers, insurance/language issues; Intervention: culturally competent PHC; Inner: staff readiness; Individuals: navigation challenges; Process: engagement, reflection	Appropriateness linked to culturally competent PHC	Language barriers, system complexity	Community health workers, patient advocates	Better service navigation and utilization
Callender et al. (2022) [54]	USA; Muslim immigrant/refugee women	Outer: discrimination, cultural stigma; Intervention: faith-based mental health; Inner: organizational support; Individuals: religiosity, self-efficacy; Process: engagement and reflection	Faith-based practices highly acceptable and feasible	Stigma, privacy concerns	Prayer, mindfulness	Stress reduction and emotional regulation
McMorrow S et al. (2017) [85]	USA Midwest; Congolese refugee women	Outer: social isolation, digital divide; Intervention: digital social platforms; Inner: supportive community culture; Individuals: digital literacy, motivation; Process: participatory design	High feasibility and adoption potential	Digital literacy gaps, limited internet	Training, community facilitation	Enhanced connectedness, reduced isolation
Baird MB et al. (2015 [86])	Unspecified; Sudanese refugee women	Outer: cultural divides, integration barriers; Intervention: community-based collaborative programs; Inner: inclusive culture; Individuals: empowerment, leadership capacity; Process: co-leadership and planning	High acceptability and sustainability with community ownership	Distrust, cultural barriers	Community co-leadership, culturally competent providers, inclusive policies, interpretation services	Greater empowerment, improved health service engagement

#### 3.4.4 End-of-life and palliative care.

Seventeen studies examined end-of-life (EOL) and palliative care interventions for refugee and displaced populations, focusing on advanced illness, symptom management, and culturally appropriate spiritual support [60,61,64,87–100].

Intervention characteristics most frequently included home-based palliative care, dignity-preserving approaches, integration of cultural and religious rituals, and cross-cultural provider training [88,95,98]. Some studies reported models linking palliative care with primary health or HIV services, which broadened access [60,87,100]. Flexible outreach and mobile teams were also described as enhancing feasibility for mobile or marginalized populations [99].

Outer Setting determinants reported as barriers included the absence of palliative care in humanitarian policy, shortages of essential medicines (particularly opioids), and legal or regulatory exclusions limiting refugee access [91,93,97]. Camp resource scarcity, unstable housing, and systemic inequities were also identified as obstacles to continuity of care [91,99]. Inner Setting constraints included limited provider preparedness, inadequate awareness of palliative approaches, and fragmented coordination between humanitarian and health actors [89,90]. Where specialist palliative units, NGO–health system partnerships, or multidisciplinary outreach teams were present, adoption and integration were stronger [61,87,90].

Characteristics of individuals influenced delivery and engagement. Provider-level skills in communication and cultural awareness were reported as shaping quality of care. Refugee patients and families frequently relied on religious rituals, faith-based coping, and cultural understandings of dignity in illness and death [88,98]. Process strategies emphasized family involvement in care planning, engagement of faith and community leaders, inclusive spatial design, and cross-sector coordination [87,89,98].

Across Proctor’s outcomes, interventions were reported as acceptable and appropriate when incorporating cultural and religious practices, family structures, and inclusive rituals [87,88]. Feasibility was reported as higher in integrated primary–palliative care models, community-based services, and flexible outreach [87,99]. Adoption was supported through NGO partnerships, community leadership, and faith-based mediation [89,98]. Fidelity was strongest when dignity-care protocols and family-inclusive planning were consistently applied. Penetration and sustainability were limited overall, though studies noted improvements when services were integrated into host-country primary care systems and supported with policy or dedicated resources [60,87].

Reported barriers included language and communication gaps, stigma, cultural misunderstandings, mistrust of formal services, shortages of trained providers, restricted opioid access, policy exclusions, and disruptions associated with displacement and legal precarity [91,93,95,97,99]. Reported facilitators included family engagement, safe and inclusive clinical environments, interpreter and mediator provision, involvement of religious leaders, and NGO–health system collaboration [87,89,98]. Reported outcomes included reductions in symptom distress and unnecessary hospitalizations [60,64,93]. Patient- and family-level outcomes included preservation of dignity, improved trust in providers, strengthened caregiver–staff relationships, and reduced emotional distress [88,93,98]. Both pediatric and adult studies reported that culturally respectful, community-integrated approaches were associated with improved comfort and increased utilization of palliative services [61,95] (Table 6).

**Table 6:** Summary of qualitative evidence on end-of-life and palliative care interventions for refugee populations mapped to CFIR constructs, Proctor’s implementation outcomes, barriers, facilitators, and reported outcomes.

Citation	Context	CFIR Components	Proctor Summary	Barriers	Facilitators	Outcomes
Wu (2015) [88]	USA; Chinese immigrants	Outer: immigrant community context; Intervention: family-centered palliative care; Inner: home-based delivery; Individuals: cultural caregiving values; Process: informal care networks	Acceptable when culturally aligned; feasibility dependent on strong family support	Language barriers, stigma around palliative care	Family involvement, incorporation of cultural rituals	Increased caregiver satisfaction
Bell (2018) [89]	Australia; refugee clinic	Outer: refugee-focused clinic; Intervention: spatial design for comfort; Inner: multidisciplinary service model; Individuals: sense of belonging; Process: participatory design	High acceptability and improved trust in care	Institutional constraints on space and resources	Inclusive design, safe and welcoming clinic spaces	Greater comfort and patient engagement
Jansky (2019) [90]	Germany; migrants	Outer: migration-related inequities; Intervention: equity-focused approach; Inner: specialist palliative units; Individuals: cultural awareness; Process: expert consultations	Acceptable if equity is addressed; sustainability requires staff training	Lack of cultural training for providers	Ongoing education, cultural mediation	Improved perceptions of equity in care
Najjar (2020) [91]	Nepal; refugee camps	Outer: camp-based living; Intervention: context-specific care; Inner: resource-limited health facilities; Individuals: cultural death rituals; Process: case reflection	Feasible when culturally adapted	Scarcity of resources, staffing shortages	Support from community volunteers	Preservation of dignity in care
Abdelaal (2021) [92]	Canada; refugee claimants	Outer: legal precarity; Intervention: case-based palliative care; Inner: urban hospital setting; Individuals: patient–provider trust; Process: case management	Acceptable when trust is established; feasible case-by-case	Policy barriers to access	Provider advocacy, flexible and tailored care	Increased patient comfort
Doherty (2020) [93]	Bangladesh; Rohingya refugees	Outer: refugee camp context; Intervention: community-integrated palliative services; Inner: field-based clinics; Individuals: trauma resilience; Process: surveys and interviews	High need; feasible with strong community linkage	Shortages of services and staff	NGO collaboration, local health worker involvement	Reduced symptom distress
Molnar (2020) [60]	Refugee health systems	Outer: national refugee health system; Intervention: integrated palliative services; Inner: primary care integration; Individuals: cultural adaptation skills; Process: policy review	Appropriate within integrated health models	Fragmented service delivery	Alignment of palliative and primary care	Improved continuity of care
De Laat (2021) [94]	Jordan & Rwanda; refugee camps	Outer: camp constraints; Intervention: collaborative service models; Inner: NGO–health sector partnerships; Individuals: cultural norms; Process: multi-stakeholder engagement	Highly appropriate; sustainability depends on partnerships	Resource and policy gaps	Cross-sector coordination and partnerships	Enhanced service coordination
de Voogd (2021) [95]	Netherlands; migrant patients	Outer: migrant community needs; Intervention: dignity-preserving care; Inner: specialist palliative units; Individuals: cultural awareness; Process: dignity-focused planning	Acceptable if dignity is central to care	Cultural misunderstandings between patients and providers	Staff training, dignity-centred approaches	Maintained patient dignity
Shabnam J et al, (2024) [96]	Denmark; migrants	Outer: migrant health needs; Intervention: equitable access; Inner: hospital-based palliative teams; Individuals: family trust; Process: interviews	Appropriateness improved when equity addressed	Language barriers	Interpreter services, inclusive policies	Increased utilisation of palliative services
Ashrafizadeh (2023) [97]	Eastern Mediterranean Region; migrants & refugees	Outer: health disparities; Intervention: culturally adapted palliative care; Inner: system capacity gaps; Individuals: provider competence; Process: regional collaboration	High appropriateness; adoption requires readiness	Policy and resource gaps	Regional partnerships, training initiatives	Improved equity in palliative service delivery
Cummins (2023) [98]	Canada; Muslim migrants	Outer: religious community structures; Intervention: faith-congruent palliative care; Inner: community–health system links; Individuals: faith-based coping; Process: narrative accounts	High acceptability with religious congruence	Stigma, mismatch between care and beliefs	Faith leader involvement, peer support	Enhanced dignity in end-of-life care
Hudson (2023) [99]	UK; homelessness & immigration status	Outer: marginalised populations; Intervention: flexible outreach palliative services; Inner: outreach teams; Individuals: complex needs; Process: adaptive planning	Acceptable when flexible and mobile	Unstable housing, mistrust of services	Mobile teams, harm reduction approaches	Increased service access
Zhang (2023) [100]	USA; HIV clinic for immigrants/refugees	Outer: urban health inequities; Intervention: HIV–palliative service linkage; Inner: multidisciplinary teams; Individuals: stigma resilience; Process: patient-centred design	Appropriate if stigma is addressed	HIV-related stigma, language barriers	Peer navigators, interpreter services	Increased uptake of palliative care
De Laat (2024) [61]	Rwanda; refugee camp	Outer: resource scarcity; Intervention: context-specific palliative models; Inner: NGO–health partnerships; Individuals: cultural practices; Process: field observation	Highly acceptable when adapted to context	Staffing and medicine shortages	NGO support, trained local health workers	Delivery of dignified care
Leng (2024) [87]	Uganda & refugee settlements	Outer: fragile health systems; Intervention: integrated primary–palliative models; Inner: primary care teams; Individuals: palliative care skills; Process: participatory appraisals	High appropriateness; feasible with integration	Service fragmentation	Joint training, leadership engagement	Strengthened integration of care
Gupta (2025) [64]	Urban hospitals serving refugees	Outer: hospital–community service gaps; Intervention: transitional palliative care; Inner: hospital units; Individuals: caregiver involvement; Process: mixed-methods	Appropriate when transitional supports are added	Bed shortages, lack of home-based services	Discharge planning, home visits	Reduced hospital-based deaths

### 3.5 Integrated CFIR–proctor synthesis of refugee health interventions

This review undertook an integrated analysis of implementation determinants, outcomes, barriers, and facilitators across the four domains of refugee health interventions. Across domains, cultural adaptation, participatory design, and intervention flexibility were consistently reported as central features shaping acceptability, appropriateness, and fidelity. For instance, stigma-sensitive and community-anchored approaches were described in mental health interventions [52,66,67], rights-based and intersectional strategies in disability inclusion [56,73,74], peer-led and co-designed delivery in women’s health [54,82,83], and dignity-preserving integration into primary health systems in palliative care [60,87,88].

When mapped to Proctor’s outcomes, findings followed a common gradient across the literature. Acceptability, appropriateness, and feasibility were the most frequently documented and were typically achieved in the early phases of implementation. By contrast, adoption, fidelity, penetration, and sustainability were reported less often and were more closely associated with interventions that demonstrated stronger systemic integration. For example, disability-inclusive education programs maintained adoption when linked to school readiness and teacher training [63], while palliative care interventions demonstrated continuity when embedded within host-country primary care structures [60, 87].

Barriers cutting across all domains were predominantly structural. Policy exclusions, regulatory gaps, stigma, discrimination, and resource scarcity including shortages of essential medicines, assistive devices, and trained personnel were widely reported as limiting penetration and scale [57,62,91,97]. These systemic constraints were compounded by logistical challenges such as unstable housing, transport difficulties, and digital divides, all of which undermined continuity and equity in service delivery [55,58,73].

Alongside these constraints, studies identified a range of facilitators that supported implementation. Participatory design processes, community and peer engagement, interpreter and cultural mediation services, cross-sector partnerships, and organizational readiness were frequently described as enabling mechanisms across domains [67,70,77,98]. Where these facilitators were present, interventions achieved stronger contextual fit and greater engagement.

The outcomes reported across domains reflected these patterns of barriers and facilitators. Documented results included improved service uptake in maternal health and preventive screening [82,84]; reductions in psychological distress, stigma, and trauma symptoms in mental health interventions [66,68]; enhanced social participation and empowerment for persons with disabilities [63,72]; and strengthened dignity, trust, and continuity of care in end-of-life and palliative interventions [88,95] (Table 7).

**Table 7:** Cross-Domain Determinants, Outcomes, Barriers, and Facilitators for Refugee Health Interventions.

Domain	Key Determinants (CFIR)	Reported Outcomes (Proctor)	Barriers & Facilitators
Mental Health	Culturally congruent psychosocial care; stigma-aware digital and faith-based tools; participatory and community-led design	High acceptability and appropriateness; improved help-seeking behavior; reduced stigma; enhanced trust in providers	**Barriers:** asylum process stress, discrimination, insecure housing, limited continuity of care. **Facilitators:** cultural adaptation, peer support networks, integration with trusted community providers
Disability Inclusion	Gender-responsive prevention protocols; adaptable developmental screening; inclusive and intersectional program design	Strong appropriateness when inclusive approaches applied; improved caregiver coping; earlier identification of disability-related needs	**Barriers:** policy and legal gaps, fragmented service coordination, entrenched stigma, inaccessible facilities. **Facilitators:** intersectional approaches, advocacy-led programming, cross-sector partnerships with NGOs and disability groups
Women’s Health	Peer-led health education; culturally tailored maternal and perinatal care; flexible digital health platforms	High adoption and feasibility; increased maternal care access; improved health literacy and continuity of care	**Barriers:** restrictive reproductive health policies, socio-economic hardship, gender norms that limit autonomy. **Facilitators:** community co-designed models, provider cultural competence, grassroots women’s engagement networks
End-of-Life/ Palliative Care	Family-centered palliation; dignity-preserving care strategies; integration into primary health care (PHC) systems	Improved appropriateness, sustainability, and satisfaction; preservation of patient dignity; reduced hospital deaths	**Barriers:** resource scarcity, unstable housing among refugees, lack of protection frameworks, fragmented specialist services. **Facilitators:** strong family involvement, trust-based provider–patient relationships, NGO–health collaborations

### 3.6 Comparative Findings by Income Setting

Of the 49 included studies, 32 were conducted primarily in high-income countries (HICs), 15 in low- and middle-income countries (LMICs), and 2 in multi-country or cross-regional settings. Mental health and disability inclusion studies were more frequently located in HIC resettlement contexts, while end-of-life and palliative care studies were more commonly situated in LMIC humanitarian settings. Women’s health studies were distributed across both income settings, though numerically fewer than studies in other domains.

Across domains, studies conducted in HICs predominantly described interventions delivered through formal health systems, including structured clinical services, interpreter-supported programs, and institutionally based models of care. Studies conducted in LMICs more often reported community-based, NGO-supported, and informal service models delivered in resource-constrained environments. A domain-specific summary of these observed differences is presented in **Table 8**.

**Table 8:** Evidence patterns and assets by income setting across four domains (n = 49).

Domain	Findings reported in HIC settings	Findings reported in LMIC settings	Primary types of assets described in HICs	Primary types of assets described in LMICs
Mental health	Studies in HIC resettlement contexts described mental health support delivered through formal services, including structured psychosocial care, use of interpreters and culturally adapted communication, and engagement with primary care or specialist pathways [59,65,66,70]. Digital or technology-enabled delivery and structured program models were also described in HIC samples, including youth-focused formats and adaptation to immigrant/refugee populations [54,55,57,71].	Studies in LMIC or humanitarian contexts described psychosocial support and coping in settings characterized by community delivery and reliance on local or humanitarian actors, including camp/settlement contexts and host-community interfaces [53,67–69]. Several LMIC studies described help-seeking and coping shaped by local social and religious practices, with support delivered outside specialist mental health systems [53,67,68].	Health-system-linked service models and provider-facing assets, including GP or provider capability, interpreter-supported communication, and organized training or learning environments [65,70,71]. Programmatic assets included structured psychosocial interventions and digital tools in HIC settings [54,55,57].	Community and social assets described as salient in LMIC settings included faith-based supports and locally embedded coping resources, with program delivery commonly reported through humanitarian or community platforms [53,67,68]. Community/NGO-linked service structures and settlement-based resources were also described [53,69].
Disability inclusion	HIC studies described barriers and service needs among resettled refugees with disabilities and chronic conditions, including navigation of health and social services and access to rehabilitation-oriented supports [62,76,79]. HIC evidence also described participation barriers and inclusion challenges in institutional settings (e.g., education or service access) and approaches to participation in research and services [63,73,77].	LMIC-focused studies reported disability-related needs and inclusion challenges in settings where services were shaped by constrained resources and community delivery, including -related prevention/response adaptations for communication disability [74]. Studies in middle-income contexts described intersectional experiences of refugee women with disabilities and community participation in host settings [56,72,78].	Formal screening and institutional mechanisms (e.g., developmental screening processes) and health-system service interfaces were described in HIC settings [76,79]. Education- and policy-adjacent inclusion assets and structured service/rehabilitation pathways were described [62,63,73].	Community-facing and adaptation-oriented assets were described, including inclusive communication approaches and community awareness strategies for communication disability (Refs: 14). Family/caregiver resources and community-based coping/support assets were described in studies involving children with disabilities and caregivers [56,72,78].
Women’s health	HIC studies described maternal/perinatal care and health navigation within established health systems, including information seeking, preventive screening experiences, and service engagement in resettlement contexts [58,81,83,84]. Evidence also described women’s experiences in accessing and using care, including perceptions shaping engagement and inequities [82,85].	LMIC and middle-income context studies described women’s health needs linked to social and health system constraints in host settings, including the health and social problems of refugee women and children and community-oriented program experiences [80]. Studies also described community-based collaborative models and women’s engagement in resource-constrained or community-led settings [53,85].	Health-system assets included structured maternity/perinatal services, preventive screening interfaces, and service navigation supports described in HIC settings [81,83,84]. Community-facing assets described within HIC contexts included peer/education-oriented approaches and information supports [58,85].	Community and social assets included community-led or collaborative approaches and local support networks described in LMIC/middle-income settings [80,86]. Community-linked delivery platforms and local participation assets were also described where women’s experiences were reported in African or camp/settlement-related contexts (Refs: [53,86].
End-of-life and palliative care	HIC evidence described palliative and end-of-life care experiences within established health systems, including provider perspectives on provision for migrant/refugee populations and caregiver experiences, with attention to communication and service organization [90,92,95,96]. Studies also described access challenges in marginal or complex legal/social situations in HIC settings [91,99,100].	LMIC and humanitarian setting studies described palliative care need and provision in camp/settlement or fragile settings, including care delivery under constrained resources and partnerships across humanitarian actors [61,87,93,94]. LMIC evidence included studies situated in refugee camps or settlement contexts with emphasis on service limits and context-shaped delivery models [53,61,87].	Institutional palliative care assets described in HICs included hospital/hospice-linked services and organized provider/caregiver interfaces [90,95,96]. Outreach or service-linkage assets for specific marginal groups were also described in HIC contexts [99,100].	Humanitarian and community-delivered palliative care assets included NGO-supported service provision in camps/settlements, partnership-based delivery models, and locally embedded caregiver/community resources [61,87,94]. Needs-oriented service assets and community linkage approaches were described in Rohingya camp-related work [93].

## 4. Discussion

This review provides a cross-domain synthesis of refugee health interventions spanning mental health, disability inclusion, women’s health, and end-of-life/palliative care. Across these domains, three implementation outcomes acceptability, appropriateness, and feasibility were most consistently achieved, while adoption, fidelity, and sustainability were observed primarily in interventions that progressed beyond pilot stages. Shared determinants of success included cultural adaptation, community engagement, participatory design, and provider cultural competence, all of which were strongly associated with higher uptake and trust. Conversely, policy gaps, stigma, discrimination, service fragmentation, and resource scarcity emerged as recurrent barriers limiting penetration and sustainability. The synthesis also identified innovative facilitators such as faith-integrated mental health models, inclusive prevention strategies, peer-led women’s health education, and family-centred palliative care, which contributed to measurable improvements in help-seeking, maternal health access, disability inclusion, and dignified end-of-life experiences. By systematically mapping determinants to implementation outcomes through a CFIR–Proctor hybrid framework, this review moves beyond domain-specific analyses to highlight transferable strategies that can inform refugee health policy and practice globally.

Findings reaffirm well-documented barriers to refugee mental health service utilization, most notably language limitations, stigma, bureaucratic hurdles, and digital access constraints, which have been consistently reported across systematic reviews [23,30,101–103]. While previous reviews have emphasized the importance of culturally sensitive care and provider training [30,101,102], few examined the operational impact of faith-based or digital modalities. The present analysis advances the literature by demonstrating how culturally congruent psychosocial models and stigma-aware digital tools can improve engagement and acceptability, linking these directly to reported reductions in depression, anxiety, and post-traumatic stress disorder [104,105].

Disability inclusion remains comparatively underrepresented in refugee health research. This review makes a novel contribution by identifying inclusive prevention models, culturally adapted developmental screening, and intersectional gender–disability programming as effective strategies. Earlier reviews have noted fragmented services and entrenched stigma as persistent barriers; however, the present synthesis identifies tangible facilitators such as advocacy-led program design, peer support networks, and teacher training. Outcomes including early identification of developmental needs and improved caregiver coping capacities provide an additional dimension of evidence seldom reported previously [106–108]. Consistent with Rfat et al. (2022), findings highlight the influence of systemic discrimination, legal restrictions, and resource scarcity in limiting access to inclusive healthcare, education, and social participation [106]. The review also aligns with calls from Njelesani et al. (2022) and Malloy et al. (2023) for participatory, accessible research design that directly involves persons with disabilities and centres their lived experiences [107,109].

In women’s health, this review confirms established evidence of refugee women’s heightened vulnerability to adverse perinatal outcomes, and mental health challenges [110], as well as the importance of social support, health literacy, and culturally responsive care [111]. While prior reviews have specifically called for improved communication and navigation [112], the present findings identify operational models including peer-led health education, participatory co-leadership, female provider engagement, and multi-sectoral service integration that were associated with improved screening uptake, interpersonal trust, and maternal mental health outcomes.

Evidence on end-of-life and palliative care for refugees remains limited, with most earlier reviews emphasizing unmet needs for culturally sensitive, family-oriented approaches but offering few empirical evaluations [113,114]. This review addresses that gap by presenting findings on family-centred palliative models, dignity-preserving care, and the integration of primary and palliative services. Whereas earlier literature highlighted the absence of structured frameworks and the challenges of delivering care in resource-constrained or unstable settings [113,115], this synthesis documents operational strategies such as outreach and mobile service models that extended access to marginalized populations. Reported outcomes included reduced symptom distress, improved dignity, and strengthened patient–provider trust, providing empirical evidence of effectiveness. These results are consistent with global calls for integrating palliative care into primary health systems, including WHO guidance on essential palliative services in humanitarian crises [116]. At the same time, most interventions remain pilot-level, lack long-term sustainability evidence, and are rarely scaled within national health strategies.

The comparative findings by income setting underscore a well-established principle in global health implementation: the effectiveness of refugee health interventions is shaped primarily by the structural and service environments in which they are delivered. Evidence from high-income countries reflects greater reliance on formal health-system assets, including specialist services, interpreter-supported care, and structured clinical pathways features that have been shown to facilitate coordination, continuity, and quality of care for refugee populations [117,118]. In contrast, studies from low- and middle-income and humanitarian settings more often described interventions implemented through community networks, non-governmental organizations, and informal support platforms operating in resource-constrained contexts, a pattern consistently documented in humanitarian mental health and psychosocial support literature [119,120]. These differences align with broader implementation science evidence demonstrating that complex health interventions achieve greater feasibility and impact when they are adapted to local system capacities and social realities rather than transferred without contextual modification [121,122]. Across domains, the present review found that cultural adaptation, participatory design, and contextual flexibility were central to acceptability and feasibility an observation consistent with extensive evidence on culturally responsive care for migrant and refugee populations [123,124]. Global policy guidance further emphasizes that community engagement and locally grounded service models are essential components of effective refugee and migrant health programming [125,126]. The predominance of studies from high-income settings nevertheless highlights a persistent imbalance in the evidence base and reinforces longstanding calls for greater investment in implementation research within low-resource and displacement-affected contexts [51]. Together, these findings point to the need for implementation strategies that prioritize contextual fit and equity, and for a rebalancing of the research agenda to better reflect the realities of the settings in which most displaced populations reside.

Taken together, the cross-domain synthesis reveals shared barriers including policy gaps, stigma, and resource scarcity and common facilitators such as cultural adaptation, community engagement, and participatory design, in line with the broader literature [23,101,103,110,111]. The unique contribution of this review lies in systematically mapping these determinants to specific implementation outcomes (acceptability, feasibility, sustainability) and demonstrating that community-driven, context-specific approaches are consistently associated with higher impact. This integrative perspective contrasts with the compartmentalized treatment of refugee health domains in prior reviews, offering a more coherent understanding of transferable strategies for donors, policymakers, and practitioners.

Methodologically, the review aligns with established scoping review frameworks (Arksey and O’Malley; Levac et al.; PRISMA-ScR), ensuring transparency, replicability, and comprehensive coverage [23,127,128]. Core processes included multi-database systematic searches, explicit eligibility criteria, and dual independent screening and data extraction [23,29,129]. While quality appraisal is not typically required in scoping reviews, the use of the CASP Qualitative Checklist enhanced interpretive validity without influencing inclusion [30]. The review also extends methodological standards by mapping findings to the CFIR framework and Proctor’s taxonomy of implementation outcomes, enabling nuanced cross-domain analysis and enhancing transferability an aspect often missing from earlier reviews [23,127,128]. The systematic integration of participatory and community-based approaches in both analysis and interpretation further distinguishes this review as methodologically robust and innovative [128].

In general, the synthesis demonstrates that community-driven, culturally adapted, and participatory models were consistently associated with higher uptake and sustainability than fragmented or top-down approaches. Shared facilitators including cultural competence, trust-based delivery, and community engagement supported implementation across all domains, while policy gaps, stigma, and resource scarcity remained persistent barriers. By aligning with global priorities articulated in the Sustainable developmental goals and WHO’s Global Action Plan for Refugee and Migrant Health, this review provides an evidence base for the design of interventions that are both locally responsive and globally transferable.

## Strengths and limitations

This review’s main strength lies in its methodological rigor and breadth. Guided by established scoping review frameworks, it used systematic multi-database searches, explicit eligibility criteria, and dual independent screening to ensure transparency and replicability. Mapping findings to CFIR and Proctor’s implementation outcomes enabled a structured cross-domain synthesis, while the inclusion of participatory and community-focused perspectives enhanced contextual relevance.

Several limitations should be acknowledged. Restricting inclusion to English-language, peer-reviewed qualitative studies may have excluded relevant non-English and grey literature, particularly from humanitarian settings. Geographic and income-level representation was uneven, with a predominance of studies from high-income resettlement contexts and comparatively limited evidence from low- and middle-income countries where most displaced populations reside. This imbalance may have led to greater documentation of formal health-system–based models and underrepresentation of community-driven approaches common in resource-constrained settings. In addition, as a scoping review, the study was not designed to assess effectiveness or establish causal relationships. Finally, findings reflect the literature available up to 2025 and may require updating as contexts and interventions evolve.

These limitations temper generalizability but do not diminish the review’s contribution in synthesizing qualitative evidence across four key domains and in identifying critical gaps and priorities for future refugee health research.

## Recommendations

Based on the mapped evidence, several recommendations can be drawn to guide future policy, practice, and research on refugee health interventions.

For policymakers, the evidence highlights the importance of integrating culturally congruent and community-driven elements into refugee health strategies. Key considerations include embedding participatory design, accommodating faith and cultural practices, and ensuring family engagement, all supported by mechanisms for cross-sector coordination and sustained financing.

For health systems and practitioners, findings underscore the need to embed culturally competent, trauma-informed, and context-specific approaches into service delivery. These efforts should be reinforced by targeted workforce training and strong partnerships with refugee communities to ensure trust, accessibility, and continuity of care. For humanitarian actors and NGOs, greater alignment with national and local health systems is critical. Priority should be given to mobile and outreach-based models of service delivery to extend coverage to underserved and mobile refugee populations who are often excluded from formal systems of care.

For researchers, there remains a need to address underexplored domains such as disability inclusion and palliative care. Future studies should expand the evidence base through participatory methodologies and mixed-methods designs, with particular attention to underrepresented geographic contexts where the majority of refugees reside.

## Conclusion

This scoping review synthesizes qualitative evidence on refugee health interventions across four priority domains mental health, disability inclusion, women’s health, and end-of-life care capturing both cross-cutting and domain-specific barriers, facilitators, and outcomes. By applying CFIR and Proctor’s frameworks, the review extends beyond descriptive mapping to identify implementation patterns that inform context-sensitive, culturally grounded, and system-integrated approaches. Although causal inferences cannot be established, the synthesis provides a robust foundation for policy development, program design, and future research. Across domains, the findings consistently emphasize the importance of community engagement, cultural adaptation, and cross-sector collaboration in improving the accessibility, quality, and sustainability of health services for displaced populations.

## Supporting information

S1 Table Search terms by database used in the scoping review. (DOCX)

S2 Table Data extraction format for included studies. (DOCX)

S3 Table Characteristics and evidence synthesis of included studies across four domains. (DOCX)

S4 Table Critical appraisal of included qualitative studies using the CASP checklist. (DOCX)

S1 Checklist PRISMA Checklist. (DOCX)

## Abbreviations

CASP: Critical Appraisal Skills Programme
HRD: Human Resource Development
IDP: Internally Displaced Person
LMIC: Low- and Middle-Income Countries
NGO: Non-Governmental Organization
PHEM: Public Health Emergency Management
GBVS: Gender-Based Violence
UNHCR: United Nations High Commissioner for Refugees
WHO: World Health Organization

## References

[1] Abubakar I, Devakumar D, Madise N, Sammonds P, Groce N, Zimmerman C, et al. UCL-Lancet Commission on Migration and Health. Lancet. 2016;388(10050):1141–2. doi: 10.1016/S0140-6736(16)31581-1 27650082

[2] Hargreaves S, Rustage K, Nellums LB, McAlpine A, Pocock N, Devakumar D, et al. Occupational health outcomes among international migrant workers: a systematic review and meta-analysis. Lancet Glob Health. 2019;7(7):e872–82. doi: 10.1016/S2214-109X(19)30204-9 31122905 PMC6565984

[3] Lindert J, Carta MG, Schäfer I, Mollica RF. Refugees mental health-A public mental health challenge. Eur J Public Health. 2016;26(3):374–5. doi: 10.1093/eurpub/ckw010 27053728

[4] Uphoff E, Robertson L, Cabieses B, Villalón FJ, Purgato M, Churchill R, et al. An overview of systematic reviews on mental health promotion, prevention, and treatment of common mental disorders for refugees, asylum seekers, and internally displaced persons. Cochrane Database Syst Rev. 2020;9(9):CD013458. doi: 10.1002/14651858.CD013458.pub2 32885850 PMC8572368

[5] Grasser LR. Addressing mental health concerns in refugees and displaced populations: is enough being done?. Risk Management and Healthcare Policy. 2022;:909–22. 35573980 10.2147/RMHP.S270233 PMC9094640

[6] Asif Z, Kienzler H. Structural barriers to refugee, asylum seeker and undocumented migrant healthcare access. Perceptions of Doctors of the World caseworkers in the UK. SSM - Mental Health. 2022;2:100088. doi: 10.1016/j.ssmmh.2022.100088

[7] Cantor D, Swartz J, Roberts B, Abbara A, Ager A, Bhutta ZA, et al. Understanding the health needs of internally displaced persons: A scoping review. J Migr Health. 2021;4:100071. doi: 10.1016/j.jmh.2021.100071 34820657 PMC8600058

[8] Ní Ghráinne B. Internally Displaced Persons and Exclusion Clauses. International Journal of Refugee Law. 2025;37(2):219–34. doi: 10.1093/ijrl/eeaf016

[9] Chuah FLH, Tan ST, Yeo J, Legido-Quigley H. The health needs and access barriers among refugees and asylum-seekers in Malaysia: a qualitative study. Int J Equity Health. 2018;17(1):120. doi: 10.1186/s12939-018-0833-x 30111329 PMC6094870

[10] El Arab RA, Somerville J, Abuadas FH, Rubinat-Arnaldo E, Sagbakken M. Health and well-being of refugees, asylum seekers, undocumented migrants, and internally displaced persons under COVID-19: a scoping review. Front Public Health. 2023;11:1145002. doi: 10.3389/fpubh.2023.1145002 37181725 PMC10169615

[11] Khanom A, Alanazy W, Couzens L, Evans BA, Fagan L, Fogarty R, et al. Asylum seekers’ and refugees’ experiences of accessing health care: a qualitative study. BJGP Open. 2021;5(6):BJGPO.2021.0059. doi: 10.3399/BJGPO.2021.0059 34376383 PMC9447303

[12] Sawadogo PM, Sia D, Onadja Y, Beogo I, Sangli G, Sawadogo N, et al. Barriers and facilitators of access to sexual and reproductive health services among migrant, internally displaced, asylum seeking and refugee women: A scoping review. PLoS One. 2023;18(9):e0291486. doi: 10.1371/journal.pone.0291486 37708137 PMC10501608

[13] Bilican S, Irfan M, Cox A, Salaets H, Sabbe M, Schoenmakers B. Access to mental healthcare for refugees, asylum seekers and migrants: an umbrella review of barriers. BMJ Open. 2025;15(6):e096267. doi: 10.1136/bmjopen-2024-096267 40484433 PMC12161349

[14] Silove D, Ventevogel P, Rees S. The contemporary refugee crisis: an overview of mental health challenges. World Psychiatry. 2017;16(2):130–9. doi: 10.1002/wps.20438 28498581 PMC5428192

[15] Warmbein A, Beiersmann C, Eulgem A, Demir J, Neuhann F. Challenges in health care services for refugees in Cologne, Germany: A providers’ perspective using a mixed-methods approach. J Migr Health. 2023;7:100158. doi: 10.1016/j.jmh.2023.100158 36866061 PMC9971550

[16] Matlin SA, Depoux A, Schütte S, Flahault A, Saso L. Migrants’ and refugees’ health: towards an agenda of solutions. Public Health Rev. 2018;39(1). doi: 10.1186/s40985-018-0104-9

[17] Khan F, Amatya B. Refugee health and rehabilitation: Challenges and response. J Rehabil Med. 2017;49(5):378–84. doi: 10.2340/16501977-2223 28440839

[18] McKeary M, Newbold B. Barriers to Care: The Challenges for Canadian Refugees and their Health Care Providers. Journal of Refugee Studies. 2010;23(4):523–45. doi: 10.1093/jrs/feq038

[19] Kwok RKH, Ho GWK. Displacement Stressors, Trauma Exposure, and Mental Health: A Survey of Asylum Seekers and Refugees. J Immigr Minor Health. 2025;27(2):208–14. doi: 10.1007/s10903-024-01668-5 39821877

[20] Saifee J, Franco-Paredes C, Lowenstein SR. Refugee Health During COVID-19 and Future Pandemics. Curr Trop Med Rep. 2021;8(3):1–4. doi: 10.1007/s40475-021-00245-2 34306967 PMC8284414

[21] Baker JR, Raman S, Kohlhoff J, George A, Kaplun C, Dadich A, et al. Optimising refugee children’s health/wellbeing in preparation for primary and secondary school: a qualitative inquiry. BMC Public Health. 2019;19(1):812. doi: 10.1186/s12889-019-7183-5 31242897 PMC6595577

[22] Selvan K, Leekha A, Abdelmeguid H, Malvankar-Mehta MS. Barriers adult refugees face to community health and patient engagement: a systematic review. Glob Public Health. 2022;17(12):3412–25. doi: 10.1080/17441692.2022.2121846 36074889

[23] Mangrio E, Sjögren Forss K. Refugees’ experiences of healthcare in the host country: a scoping review. BMC Health Serv Res. 2017;17(1):814. doi: 10.1186/s12913-017-2731-0 29216876 PMC5721651

[24] P Iqbal M, Walpola R, Harris-Roxas B, Li J, Mears S, Hall J, et al. Improving primary health care quality for refugees and asylum seekers: A systematic review of interventional approaches. Health Expect. 2022;25(5):2065–94. doi: 10.1111/hex.13365 34651378 PMC9615090

[25] Watters C. Emerging paradigms in the mental health care of refugees. Soc Sci Med. 2001;52(11):1709–18. doi: 10.1016/s0277-9536(00)00284-7 11327142

[26] Frounfelker RL, Miconi D, Farrar J, Brooks MA, Rousseau C, Betancourt TS. Mental Health of Refugee Children and Youth: Epidemiology, Interventions, and Future Directions. Annu Rev Public Health. 2020;41:159–76. doi: 10.1146/annurev-publhealth-040119-094230 31910713 PMC9307067

[27] Munich O b L-M-U, Unger C p H v. Workshop: Participatory health research with refugees: Voices, experiences, and methodologies. European Journal of Public Health. 2021;31(Supplement_3):ckab164.

[28] Larsen A, Guler J. Digital Mental Health Approaches to Improve Well-Being of Refugee Families. Psychiatr Serv. 2024;75(2):198–201. doi: 10.1176/appi.ps.20230179 37554001

[29] Nowak AC, Namer Y, Hornberg C. Health Care for Refugees in Europe: A Scoping Review. Int J Environ Res Public Health. 2022;19(3):1278. doi: 10.3390/ijerph19031278 35162300 PMC8834962

[30] Satinsky E, Fuhr DC, Woodward A, Sondorp E, Roberts B. Mental health care utilisation and access among refugees and asylum seekers in Europe: A systematic review. Health Policy. 2019;123(9):851–63. doi: 10.1016/j.healthpol.2019.02.007 30850148

[31] Elnakib S, Jackson C, Lalani U, Shawar YR, Bennett S. How integration of refugees into national health systems became a global priority: a qualitative policy analysis. Confl Health. 2024;18(Suppl 1):31. doi: 10.1186/s13031-024-00587-4 38622721 PMC11017473

[32] Olcoń K, Rambaldini-Gooding D, Degeling C. Implementation gaps in culturally responsive care for refugee and migrant maternal health in New South Wales, Australia. BMC Health Serv Res. 2023;23(1):42. doi: 10.1186/s12913-023-09066-7 36650536 PMC9843667

[33] Reynolds CW, Rha JY, Lenselink AM, Asokumar D, Zebib L, Rana GK, et al. Innovative strategies and implementation science approaches for health delivery among migrants in humanitarian settings: A scoping review. PLOS Glob Public Health. 2024;4(12):e0003514. doi: 10.1371/journal.pgph.0003514 39621734 PMC11611092

[34] Yelland J, Riggs E, Szwarc J, Casey S, Dawson W, Vanpraag D, et al. Bridging the Gap: using an interrupted time series design to evaluate systems reform addressing refugee maternal and child health inequalities. Implement Sci. 2015;10:62. doi: 10.1186/s13012-015-0251-z 25924721 PMC4425879

[35] Ho S, Javadi D, Causevic S, Langlois EV, Friberg P, Tomson G. Intersectoral and integrated approaches in achieving the right to health for refugees on resettlement: a scoping review. BMJ Open. 2019;9(7):e029407. doi: 10.1136/bmjopen-2019-029407 31266840 PMC6609038

[36] Harjani MG, Stathakarou N, Konstantinidis ST, Dratsiou I, Varella A, Salcedo VT, et al. Identifying the Health Educational Needs of Refugees: Empirical Evidence from a Delphi Study. J Immigr Minor Health. 2024;26(6):984–97. doi: 10.1007/s10903-024-01626-1 39237850 PMC11607020

[37] O’Brien BC, Harris IB, Beckman TJ, Reed DA, Cook DA. Standards for reporting qualitative research: a synthesis of recommendations. Acad Med. 2014;89(9):1245–51. doi: 10.1097/ACM.0000000000000388 24979285

[38] Miller KE, Rasmussen A. War exposure, daily stressors, and mental health in conflict and post-conflict settings: bridging the divide between trauma-focused and psychosocial frameworks. Soc Sci Med. 2010;70(1):7–16. doi: 10.1016/j.socscimed.2009.09.029 19854552

[39] Lewin S. Qualitative Evidence Synthesis (QES) for Guidelines: Paper 2 - Using qualitative evidence synthesis findings to inform evidence-to-decision frameworks and recommendations. Health Res Policy Syst. 2019;17(1):75. 10.1186/s12961-019-0468-4 PMC6686513 31391119

[40] Tong A, Flemming K, McInnes E, Oliver S, Craig J. Enhancing transparency in reporting the synthesis of qualitative research: ENTREQ. BMC Med Res Methodol. 2012;12:181. doi: 10.1186/1471-2288-12-181 23185978 PMC3552766

[41] Peters M, et al. Guidance for the conduct of JBI scoping reviews chapter 11: Scoping reviews scoping reviews. Underst scoping Rev Defin Purp Process. 2017;18(10):2119–26. 10.11124/JBIES-20-00167 33038124

[42] Arksey H, O’Malley L. Scoping studies: towards a methodological framework. International Journal of Social Research Methodology. 2005;8(1):19–32. doi: 10.1080/1364557032000119616

[43] Levac D, Colquhoun H, O’Brien KK. Scoping studies: advancing the methodology. Implement Sci. 2010;5:69. doi: 10.1186/1748-5908-5-69 20854677 PMC2954944

[44] Tricco AC, Lillie E, Zarin W, O’Brien KK, Colquhoun H, Levac D, et al. PRISMA Extension for Scoping Reviews (PRISMA-ScR): Checklist and Explanation. Ann Intern Med. 2018;169(7):467–73. doi: 10.7326/M18-0850 30178033

[45] McHugh ML. Interrater reliability: the kappa statistic. Biochem Med (Zagreb). 2012;22(3):276–82. doi: 10.11613/bm.2012.031 23092060 PMC3900052

[46] Landis JR, Koch GG. The measurement of observer agreement for categorical data. Biometrics. 1977;33(1):159–74. doi: 10.2307/2529310 843571

[47] Thomas J, Harden A. Methods for the thematic synthesis of qualitative research in systematic reviews. BMC Med Res Methodol. 2008;8:45. doi: 10.1186/1471-2288-8-45 18616818 PMC2478656

[48] Kirk MA, Kelley C, Yankey N, Birken SA, Abadie B, Damschroder L. A systematic review of the use of the Consolidated Framework for Implementation Research. Implement Sci. 2016;11:72. doi: 10.1186/s13012-016-0437-z 27189233 PMC4869309

[49] Proctor E, Silmere H, Raghavan R, Hovmand P, Aarons G, Bunger A, et al. Outcomes for implementation research: conceptual distinctions, measurement challenges, and research agenda. Adm Policy Ment Health. 2011;38(2):65–76. doi: 10.1007/s10488-010-0319-7 20957426 PMC3068522

[50] CASP U. Critical appraisal skills programme (CASP). Qualitative checklist. CASP Oxford. 2018.

[51] Peters MD, et al. Scoping reviews. JBI manual for evidence synthesis. 2020.

[52] Walther L, Amann J, Flick U, Ta TMT, Bajbouj M, Hahn E. A qualitative study on resilience in adult refugees in Germany. BMC Public Health. 2021;21(1):828. doi: 10.1186/s12889-021-10817-6 33931077 PMC8086291

[53] Robinson J, Chiumento A, Kasujja R, Rutayisire T, White R. The “good life”, personal appearance, and mental health of Congolese refugees in Rwanda and Uganda. Soc Sci Med. 2022;293:114641. doi: 10.1016/j.socscimed.2021.114641 34922041

[54] Callender KA, Ong LZ, Othman EH. Prayers and Mindfulness in Relation to Mental Health among First-Generation Immigrant and Refugee Muslim Women in the USA: An Exploratory Study. J Relig Health. 2022;61(5):3637–54. doi: 10.1007/s10943-022-01600-x 35748969 PMC9243984

[55] Fabian KE, Turner M, Foster KT, Chwastiak L, Wagenaar BH. Pilot Testing a Digital Transdiagnostic Mental Health Intervention for Use Among Immigrant and Refugee Youth in the USA: A Mixed Methods Evaluation of Clinical and Implementation Outcomes. J technol behav sci. 2025;10(3):624–36. doi: 10.1007/s41347-024-00471-1

[56] Karadag Avci Y, Sengul I. Navigating Intersectional Complexities: A Narrative Analysis of Syrian Refugee Women With Disabilities in Turkey. SI. 2024;12. doi: 10.17645/si.8772

[57] Khan BM, Waserman J, Patel M. Perspectives of Refugee Youth Experiencing Homelessness: A Qualitative Study of Factors Impacting Mental Health and Resilience. Front Psychiatry. 2022;13:917200. doi: 10.3389/fpsyt.2022.917200 35747095 PMC9211750

[58] Griffin G, Nau SZ, Ali M, Riggs E, Dantas JAR. Seeking Health Information: A Qualitative Study of the Experiences of Women of Refugee Background from Myanmar in Perth, Western Australia. Int J Environ Res Public Health. 2022;19(6):3289. doi: 10.3390/ijerph19063289 35328976 PMC8951186

[59] Shannon PJ, Wieling E, McCleary JS, Becher E. Exploring the mental health effects of political trauma with newly arrived refugees. Qual Health Res. 2015;25(4):443–57. doi: 10.1177/1049732314549475 25185161

[60] Molnar A, Isaac M. Palliative and end-of-life care. Refugee health care: an essential medical guide. 2020. p. 181–92.

[61] de Laat S, Musoni ER, Bezanson K, Yantzi R, Wahoush O, Nouvet E, et al. They do their utmost: promise and limits of palliative care in two refugee camps in Rwanda, a qualitative study. Med Confl Surviv. 2024;40(2):153–81. doi: 10.1080/13623699.2024.2339732 38634428

[62] Mirza M, Heinemann AW. Service needs and service gaps among refugees with disabilities resettled in the United States. Disabil Rehabil. 2012;34(7):542–52. doi: 10.3109/09638288.2011.611211 21981065

[63] Bacakova M. Educational Hospitality at the Intersection of Forced Migration and Disability: Listening to the Voices of Ukrainian Refugee Children with Disabilities living in Germany. EJIE. 2025;4(1):20–30. doi: 10.7146/ejie.v4i1.149584

[64] Gupta P, Gill A, Panza M, Wahoush O, Saeed H, Chagani JA, et al. “'We don’t want them to have to live out their lives in the hospital”: mixed-methods study exploring palliative care needs amongst refugees’. Palliat Care Soc Pract. 2025;19:26323524251317539. doi: 10.1177/26323524251317539 39926420 PMC11803610

[65] Kienzler H. Community integration, quality of life, thriving, and mental health among refugees and asylum seekers. A London service provider perspective. Front Public Health. 2024;12:1358250. doi: 10.3389/fpubh.2024.1358250 38699416 PMC11063373

[66] Paudyal P, Tattan M, Cooper MJF. Qualitative study on mental health and well-being of Syrian refugees and their coping mechanisms towards integration in the UK. BMJ Open. 2021;11(8):e046065. doi: 10.1136/bmjopen-2020-046065 34417211 PMC8381320

[67] Al Laham D, Ali E, Mousally K, Nahas N, Alameddine A, Venables E. Perceptions and Health-Seeking Behaviour for Mental Illness Among Syrian Refugees and Lebanese Community Members in Wadi Khaled, North Lebanon: A Qualitative Study. Community Ment Health J. 2020;56(5):875–84. doi: 10.1007/s10597-020-00551-5 31965411 PMC7250961

[68] Bridi L, Kaki DA, Behnam R, Khan X, Albahsahli B, Bencheikh N, et al. Attitudes toward dementia and cognitive aging among Syrian refugees resettled in Jordan: a qualitative study. BMC Public Health. 2023;23(1):2307. doi: 10.1186/s12889-023-17183-5 37990313 PMC10664261

[69] Ahmed Z, Crush J, Owuor S, Onyango EO. Disparities and determinants of Somali refugee food security in Nairobi, Kenya. Global Food Security. 2024;43:100808. doi: 10.1016/j.gfs.2024.100808

[70] Jensen NK, Norredam M, Priebe S, Krasnik A. How do general practitioners experience providing care to refugees with mental health problems? A qualitative study from Denmark. BMC Fam Pract. 2013;14:17. doi: 10.1186/1471-2296-14-17 23356401 PMC3568406

[71] Silver C, Williams S, Forty L. Cultural competency and mental health training for medical students: Learning from refugees and asylum seekers. Health Education Journal. 2023;82(6):708–21. doi: 10.1177/00178969231182104

[72] Scheer S, Mondaca M. Unseen abilities – how refugee women with disabilities experience social participation. European Journal of Public Health. 2022;32(Supplement_3). doi: 10.1093/eurpub/ckac129.605

[73] Tofani M, Iorio S, Berardi A, Galeoto G, Conte A, Fabbrini G, et al. Disability, Rehabilitation, and Assistive Technologies for Refugees and Asylum Seekers in Italy: Policies and Challenges. Societies. 2023;13(3):63. doi: 10.3390/soc13030063

[74] Marshall J, Barrett H. Prevention of, and response to, sexual and gender-based violence, for refugees who experience communication disability: Evidence from Rwanda. Int J Speech Lang Pathol. 2025;:1–17. doi: 10.1080/17549507.2025.2484313 40237638

[75] Serrano S, Martin D. Immigration, Disability and Healthcare Access in Brazil. DSQ. 2021;41(2). doi: 10.18061/dsq.v41i2.7501

[76] Kroening ALH, Moore JA, Welch TR, Halterman JS, Hyman SL. Developmental Screening of Refugees: A Qualitative Study. Pediatrics. 2016;138(3):e20160234. doi: 10.1542/peds.2016-0234 27527798 PMC5005020

[77] Harris J, Roberts K. Challenging Barriers to Participation in Qualitative Research: Involving Disabled Refugees. International Journal of Qualitative Methods. 2003;2(2):14–22. doi: 10.1177/160940690300200202

[78] Fayad Z, Bakhsh HR, AlHeresh R. Refugee Caregivers’ Perceptions of Using Mindfulness-Based Interventions to Support Coping Skills in Children with Disability in Jordan. Children (Basel). 2024;11(11):1381. doi: 10.3390/children11111381 39594956 PMC11592726

[79] Mirza M, Luna R, Mathews B, Hasnain R, Hebert E, Niebauer A, et al. Barriers to healthcare access among refugees with disabilities and chronic health conditions resettled in the US Midwest. J Immigr Minor Health. 2014;16(4):733–42. doi: 10.1007/s10903-013-9906-5 24052476

[80] Adibelli D, Şahan Ö. Health and Social Problems of Refugee Women and Their Children: A Qualitative Case Study. Public Health Nurs. 2025;42(3):1226–34. doi: 10.1111/phn.13520 39780374

[81] Woodgate RL, Busolo DS, Crockett M, Dean RA, Amaladas MR, Plourde PJ. A qualitative study on African immigrant and refugee families’ experiences of accessing primary health care services in Manitoba, Canada: it’s not easy!. Int J Equity Health. 2017;16(1):5. doi: 10.1186/s12939-016-0510-x 28068998 PMC5223444

[82] Kasper A, Mohwinkel L-M, Nowak AC, Kolip P. Maternal health care for refugee women - A qualitative review. Midwifery. 2022;104:103157. doi: 10.1016/j.midw.2021.103157 34736016

[83] Due C, Walsh M, Aldam I, Winter A, Cooper S, Sheriff J, et al. Perinatal care for women with refugee backgrounds from African countries: a qualitative study of intersections with psychological wellbeing. BMC Pregnancy Childbirth. 2022;22(1):628. doi: 10.1186/s12884-022-04957-9 35941567 PMC9358632

[84] Babatunde-Sowole OO, Power T, Davidson PM, DiGiacomo M, Jackson D. Health screening and preventative health care in refugee women: A qualitative analysis. Contemp Nurse. 2020;56(1):62–79. doi: 10.1080/10376178.2020.1739543 32141400

[85] McMorrow S, Saksena J. Voices and Views of Congolese Refugee Women: A Qualitative Exploration to Inform Health Promotion and Reduce Inequities. Health Educ Behav. 2017;44(5):769–80. doi: 10.1177/1090198117726572 28868929

[86] Baird MB, Domian EW, Mulcahy ER, Mabior R, Jemutai-Tanui G, Filippi MK. Creating a Bridge of Understanding between Two Worlds: Community-Based Collaborative-Action Research with Sudanese Refugee Women. Public Health Nurs. 2015;32(5):388–96. doi: 10.1111/phn.12172 25572485

[87] Leng M, Downing J, Purewal G, Namukwaya L, Opia V, Venkateswaran C, et al. Evaluation of the integration of palliative care in a fragile setting amongst host and refugee communities: Using consecutive rapid participatory appraisals. Palliat Med. 2024;38(8):818–29. doi: 10.1177/02692163241269129 39248127

[88] Wu H. Palliative and end-of-life care for Chinese immigrants: experiences of family caregivers. Memorial University of Newfoundland. 2015.

[89] Bell SE. Placing care: embodying architecture in hospital clinics for immigrant and refugee patients. Materialities of care: Encountering health and illness through artefacts and architecture. 2018. p. 72–83. 10.1111/1467-9566.12604 29464770

[90] Jansky M, Owusu-Boakye S, Nauck F. “An odyssey without receiving proper care” - experts’ views on palliative care provision for patients with migration background in Germany. BMC Palliat Care. 2019;18(1):8. doi: 10.1186/s12904-019-0392-y 30665379 PMC6341678

[91] Najjar SN, Hauck FR. Challenges in the Provision of End-of-Life and Palliative Care to Ethnic Nepali Refugees. J Pain Symptom Manage. 2020;60(2):476–86. doi: 10.1016/j.jpainsymman.2020.03.011 32205134

[92] Abdelaal M, Blake C, Lau J. Challenges of Providing Palliative and End-of-Life Care to Refugee Claimants in Canada: A Case Report. J Palliat Med. 2021;24(4):635–8. doi: 10.1089/jpm.2020.0422 33196336

[93] Doherty M, Power L, Petrova M, Gunn S, Powell R, Coghlan R, et al. Illness-related suffering and need for palliative care in Rohingya refugees and caregivers in Bangladesh: A cross-sectional study. PLoS Med. 2020;17(3):e1003011. doi: 10.1371/journal.pmed.1003011 32126076 PMC7053708

[94] de Laat S, Wahoush O, Jaber R, Khater W, Musoni E, Abu Siam I, et al. A case analysis of partnered research on palliative care for refugees in Jordan and Rwanda. Confl Health. 2021;15(1):2. doi: 10.1186/s13031-020-00333-6 33407734 PMC7789221

[95] de Voogd X, Willems DL, Torensma M, Onwuteaka-Philipsen BD, Suurmond JL. Dignity of informal caregivers of migrant patients in the last phase of life: a qualitative study. BMC Palliat Care. 2021;20(1):26. doi: 10.1186/s12904-021-00721-6 33541334 PMC7863486

[96] Shabnam J, Timm HU, Nielsen DS, Raunkiær M. Palliative Care Utilisation Among Non-Western Migrants in Denmark: A Qualitative Study Of the Experiences of Patients, Family Caregivers and Healthcare Professionals. Omega (Westport). 2024;90(2):805–33. doi: 10.1177/00302228221111933 35786059

[97] Ashrafizadeh H, Rassouli M. Addressing health disparities: Palliative care for migrants and refugees in the Eastern Mediterranean Region. Asia Pac J Oncol Nurs. 2023;10(12):100318. doi: 10.1016/j.apjon.2023.100318 38076295 PMC10709009

[98] Cummins A, Dossa P a r i n. Social palliation: Canadian Muslims’ storied lives on living and dying. Anthropologica. 2023;65(1):1–4.

[99] Hudson BF, Dzeng E, Burnett A, Yeung M, Shulman C. Palliative care, homelessness, and restricted or uncertain immigration status. Palliat Care Soc Pract. 2023;17:26323524231216993. doi: 10.1177/26323524231216993 38144973 PMC10748891

[100] Zhang S, Altomare A, Zuckerman RA. Understanding care experiences amongst immigrant and refugee clients in a Ryan-White funded HIV clinic: Insights to improve culturally competent care. Open Forum Infectious Diseases. 2023. 10.1038/s43856-025-00879-7 PMC12152174 40494950

[101] DeSa S, Gebremeskel AT, Omonaiye O, Yaya S. Barriers and facilitators to access mental health services among refugee women in high-income countries: a systematic review. Syst Rev. 2022;11(1):62. doi: 10.1186/s13643-022-01936-1 35387680 PMC8985267

[102] Tahir R, Due C, Ward P, Ziersch A. Understanding mental health from the perception of Middle Eastern refugee women: A critical systematic review. SSM - Mental Health. 2022;2:100130. doi: 10.1016/j.ssmmh.2022.100130

[103] Dumke L, Wilker S, Hecker T, Neuner F. Barriers to accessing mental health care for refugees and asylum seekers in high-income countries: A scoping review of reviews mapping demand and supply-side factors onto a conceptual framework. Clin Psychol Rev. 2024;113:102491. doi: 10.1016/j.cpr.2024.102491 39213812

[104] Fadhlia TN, Doosje B, Sauter DA. The Socio-Ecological Factors Associated with Mental Health Problems and Resilience in Refugees: A Systematic Scoping Review. Trauma Violence Abuse. 2025;26(3):598–616. doi: 10.1177/15248380241284594 39377543 PMC12145474

[105] Gruner D, Magwood O, Bair L, Duff L, Adel S, Pottie K. Understanding Supporting and Hindering Factors in Community-Based Psychotherapy for Refugees: A Realist-Informed Systematic Review. Int J Environ Res Public Health. 2020;17(13):4618. doi: 10.3390/ijerph17134618 32604990 PMC7369747

[106] Rfat M, Zeng Y, Yang Y, Adhikari K, Zhu Y. A Scoping Review of Needs and Barriers to Achieving A Livable Life among Refugees with Disabilities: Implications for Future Research, Practice, and Policy. Journal of Evidence-Based Social Work. 2022;20(3):373–403. doi: 10.1080/26408066.2022.2162357

[107] Malloy A, Rogers CM, Caine V, Clandinin DJ. Experiences of refugee children living with disabilities: a systematic review. IJMHSC. 2023;19(2):108–21. doi: 10.1108/ijmhsc-06-2022-0058

[108] Markey K, et al. Enablers of and barriers to perinatal mental healthcare access and healthcare provision for refugee and asylum-seeking women in the WHO European region: A scoping review. Healthcare. 2024. 10.3390/healthcare12171742 PMC11395031 39273766

[109] Njelesani J, Mlambo V, Denekew T, Hunleth J. Inclusion of children with disabilities in qualitative health research: A scoping review. PLoS One. 2022;17(9):e0273784. doi: 10.1371/journal.pone.0273784 36048816 PMC9436059

[110] Hawkins MM, Schmitt ME, Adebayo CT, Weitzel J, Olukotun O, Christensen AM, et al. Promoting the health of refugee women: a scoping literature review incorporating the social ecological model. Int J Equity Health. 2021;20(1):45. doi: 10.1186/s12939-021-01387-5 33485342 PMC7825239

[111] Rogers HJ, Hogan L, Coates D, Homer CSE, Henry A. Responding to the health needs of women from migrant and refugee backgrounds-Models of maternity and postpartum care in high-income countries: A systematic scoping review. Health Soc Care Community. 2020;28(5):1343–65. doi: 10.1111/hsc.12950 31997461

[112] Heslehurst N, Brown H, Pemu A, Coleman H, Rankin J. Perinatal health outcomes and care among asylum seekers and refugees: a systematic review of systematic reviews. BMC Med. 2018;16(1):89. doi: 10.1186/s12916-018-1064-0 29890984 PMC5996508

[113] Madi F, Ismail H, Fouad FM, Kerbage H, Zaman S, Jayawickrama J, et al. Death, Dying, and End-of-Life Experiences Among Refugees: A Scoping Review. J Palliat Care. 2019;34(2):139–44. doi: 10.1177/0825859718812770 30458699

[114] Nouvet E, Sivaram M, Bezanson K, Krishnaraj G, Hunt M, de Laat S, et al. Palliative care in humanitarian crises: a review of the literature. Int J Humanitarian Action. 2018;3(1). doi: 10.1186/s41018-018-0033-8

[115] Care IP. Symptom relief into the response to humanitarian emergencies and crises: a WHO guide. Geneva: World Health Organization. 2018.

[116] Schwartz L, Nouvet E, de Laat S, Yantzi R, Wahoush O, Khater WA, et al. Aid when “there is nothing left to offer”: Experiences of palliative care and palliative care needs in humanitarian crises. PLOS Glob Public Health. 2023;3(2):e0001306. doi: 10.1371/journal.pgph.0001306 36962993 PMC10021221

[117] Betancourt TS, Chambers DA. Optimizing an Era of Global Mental Health Implementation Science. JAMA Psychiatry. 2016;73(2):99–100. doi: 10.1001/jamapsychiatry.2015.2705 26720304

[118] Murray K, Davidson G, Schweitzer R. Psychological wellbeing of refugees resettling in Australia: A literature review prepared for the Australian Psychological Society. 2008.

[119] Ventevogel P. Mental health and psychosocial support for refugees, asylum seekers and migrants on the move in Europe: A multi-agency guidance note. Geneva: World Health Organization. 2015.

[120] Tol WA, Barbui C, Galappatti A, Silove D, Betancourt TS, Souza R, et al. Mental health and psychosocial support in humanitarian settings: linking practice and research. Lancet. 2011;378(9802):1581–91. doi: 10.1016/S0140-6736(11)61094-5 22008428 PMC3985411

[121] Craig P, Dieppe P, Macintyre S, Michie S, Nazareth I, Petticrew M, et al. Developing and evaluating complex interventions: the new Medical Research Council guidance. BMJ. 2008;337:a1655. doi: 10.1136/bmj.a1655 18824488 PMC2769032

[122] Proctor E, Silmere H, Raghavan R, Hovmand P, Aarons G, Bunger A, et al. Outcomes for implementation research: conceptual distinctions, measurement challenges, and research agenda. Adm Policy Ment Health. 2011;38(2):65–76. doi: 10.1007/s10488-010-0319-7 20957426 PMC3068522

[123] Kirmayer LJ, Narasiah L, Munoz M, Rashid M, Ryder AG, Guzder J, et al. Common mental health problems in immigrants and refugees: general approach in primary care. CMAJ. 2011;183(12):E959-67. doi: 10.1503/cmaj.090292 20603342 PMC3168672

[124] Citaristi I. United Nations High Commissioner for Refugees—UNHCR. The Europa Directory of International Organizations 2022. Routledge. 2022. p. 220–40. doi: 10.4324/9781003292548-50

[125] Organization WH. World report on the health of refugees and migrants: summary. World Health Organization. 2022.

[126] Committee IAS. IASC guidelines on mental health and psychosocial support in emergency settings. Geneva, Switzerland: IASC. 2006.

[127] Filler T, Benipal PK, Torabi N, Minhas RS. A chair at the table: a scoping review of the participation of refugees in community-based participatory research in healthcare. Global Health. 2021;17(1):103. doi: 10.1186/s12992-021-00756-7 34488810 PMC8420006

[128] Ungar M, Seymour A. Access Without Borders: A Scoping Review to Identify Solutions to Creating Portable Identity, Education and Health Records for Refugee Children. Int Migration & Integration. 2024;25(4):1989–2017. doi: 10.1007/s12134-024-01156-7

[129] Kocot E, Szetela A. Assessing health systems’ preparedness for providing care for refugees, asylum seekers and migrants: a scoping review. Eur J Public Health. 2020;30(6):1157–63. doi: 10.1093/eurpub/ckaa135 32840304 PMC7733048

