# Supplementary material for: Community health assets and refugee wellbeing: Qualitative evidence across mental health, disability inclusion, end-of-life care, and women’s health – A global scoping review
Source: PLOS Glob Public Health. 2026 Feb 20;6(2):e0005459. doi: 10.1371/journal.pgph.0005459 (PMC12923035; doi:10.1371/journal.pgph.0005459)
Supplement: S1 Table — (DOCX) [file pgph.0005459.s001.docx]

### S1 Table . Search Terms by Database Used in the Scoping Review of Refugee Health Interventions

| **Database** | **Search Terms** | **Number of articles identified** |
| --- | --- | --- |
| **PubMed** | (("refugee"[Title/Abstract] OR "asylum seeker"[Title/Abstract] OR "forced migrant"[Title/Abstract] OR "displaced person"[Title/Abstract] OR "internally displaced person"[Title/Abstract] OR "IDP"[Title/Abstract] OR "stateless person"[Title/Abstract]) AND ("mental health"[Title/Abstract] OR "psychosocial"[Title/Abstract] OR "PTSD"[Title/Abstract] OR "depression"[Title/Abstract] OR "anxiety"[Title/Abstract] OR "psychological distress"[Title/Abstract] OR "wellbeing"[Title/Abstract] OR "MHPSS"[Title/Abstract] OR "disability"[Title/Abstract] OR "impairment"[Title/Abstract] OR "developmental delay"[Title/Abstract] OR "intellectual disability"[Title/Abstract] OR "physical disability"[Title/Abstract] OR "women's health"[Title/Abstract] OR "maternal health"[Title/Abstract] OR "reproductive health"[Title/Abstract] OR "perinatal health"[Title/Abstract] OR "antenatal care"[Title/Abstract] OR "postnatal care"[Title/Abstract] OR "gender-based violence"[Title/Abstract] OR "GBV"[Title/Abstract] OR "intimate partner violence"[Title/Abstract] OR "end of life"[Title/Abstract] OR "palliative care"[Title/Abstract] OR "terminal care"[Title/Abstract] OR "pain management"[Title/Abstract] OR "symptom management"[Title/Abstract]) AND ("qualitative research"[Title/Abstract] OR "qualitative"[Title/Abstract] OR "interview*"[Title/Abstract] OR "focus group*"[Title/Abstract] OR "ethnography*"[Title/Abstract] OR "phenomenology*"[Title/Abstract] OR "grounded theory"[Title/Abstract] OR "case study"[Title/Abstract] OR "thematic analysis"[Title/Abstract] OR "framework analysis"[Title/Abstract] OR "content analysis"[Title/Abstract]) AND ("health services"[Title/Abstract] OR "health system"[Title/Abstract] OR "primary health care"[Title/Abstract] OR "public health"[Title/Abstract] OR "community health"[Title/Abstract] OR "healthcare access"[Title/Abstract] OR "humanitarian"[Title/Abstract] OR "humanitarian setting"[Title/Abstract] OR "humanitarian crisis"[Title/Abstract] OR "conflict-affected"[Title/Abstract])) AND ((ffrft[Filter]) AND (humans[Filter]) AND (english[Filter])) | 277 |
| **Scopus** | TITLE-ABS-KEY (refugee* OR "asylum seeker*" OR "displaced person*" OR "forced migrant*" OR "internally displaced person*" OR IDP OR "stateless person*" OR "humanitarian migrant*" OR "forced displacement" OR "migrant population*" OR "forcibly displaced population*")  AND TITLE-ABS-KEY ( "mental health" OR "psychosocial support" OR trauma OR PTSD OR "post-traumatic stress disorder" )AND TITLE-ABS-KEY ( qualitative* OR interview* OR "focus group*" OR ethnograph* ) | 1809 |
| **Web of Science** | TS=("refugee*" OR "asylum seeker*" OR "displaced person*" OR "forced migrant*" OR "internally displaced person*" OR "IDP" OR "stateless person*" OR "forcibly displaced population*") AND  TS=("mental health" OR "psychosocial support" OR "trauma" OR "PTSD" OR "psychological distress" OR "disability" OR "disabled persons" OR "impairment" OR "rehabilitation" OR "end-of-life care" OR "palliative care" OR "terminal illness" OR "advance care planning" OR "maternal health" OR "women's health" OR "perinatal care" OR "reproductive health") AND TS=("qualitative" OR "interview*" OR "focus group*" OR "ethnograph*" OR "narrative" OR "thematic analysis" OR "grounded theory" OR "phenomenolog*") | 206 |
| **Google scholar** | allintitle: ("refugee health") AND qualitative AND ("mental health" OR disability OR "women's health" OR palliative) after:2015 | 6 |
| **WHO-IRIS** | "Refugee health” AND ("mental health" OR "disability" OR "women's health" OR "palliative care”) AND qualitative AND (implementation OR "program evaluation" OR "community engagement") | 243 |
| **UNHCR** | site: unhcr.org "refugee health" AND qualitative AND (implementation OR "program evaluation") filetype:pdf after:2015 | 24 |
| **IOM** | site:iom.int "refugee health" qualitative AND "evaluation report" filetype:pdf after:2015 | 1 |
| **UNICEF** | site:unicef.org ("refugee health"AND " health" AND qualitative AND (program evaluation" ) filetype:pdf after:2015 | 2 |
| **MSF** | site: msf.org ("refugee health" AND qualitative AND (implementation OR "program evaluation" OR "community engagement") AND (report OR guideline OR framework OR policy) filetype:pdf after:2015 | 6 |
| **Safe the Children** | site: savethechildren.org ("refugee health" AND qualitative AND (implementation OR "program evaluation" OR "community engagement") AND (report OR guideline OR framework OR policy) filetype:pdf after:2015 | 3 |
