# Supplementary material for: Community health assets and refugee wellbeing: Qualitative evidence across mental health, disability inclusion, end-of-life care, and women’s health – A global scoping review
Source: PLOS Glob Public Health. 2026 Feb 20;6(2):e0005459. doi: 10.1371/journal.pgph.0005459 (PMC12923035; doi:10.1371/journal.pgph.0005459)
Supplement: S2 Table — (DOCX) [file pgph.0005459.s002.docx]

**S2 Table. Data Extraction Format for Included Studies**

| **Variable** | **Description** |
| --- | --- |
| Reference | Authors and year of publication used to identify and cite each included study |
| Country / Setting | Geographic location and study context, including country, camp-based settings, urban resettlement environments, or other relevant implementation contexts |
| Domain | The primary thematic focus of the study, classified as Mental Health, Disability Inclusion, Women’s Health, or End-of-Life and Palliative Care |
| Primary Population | Main participant group(s) targeted in the study, such as refugees, asylum seekers, internally displaced persons, or mixed displaced populations |
| Age | Age group(s) of participants as reported by study authors, including children, adolescents, adults, older adults, or mixed-age samples |
| Gender | Gender composition of participants, recorded as women-only, men-only, mixed-gender, or not reported |
| Pertinent Findings | Key qualitative findings relevant to the review objectives, including descriptions of experiences, interventions, barriers, facilitators, and contextual influences |
| Limitations | Methodological, contextual, or practical limitations explicitly acknowledged by study authors |
| Recommendations | Actionable recommendations proposed by study authors or institutions based on study findings |
