# Supplementary material for: Community health assets and refugee wellbeing: Qualitative evidence across mental health, disability inclusion, end-of-life care, and women’s health – A global scoping review
Source: PLOS Glob Public Health. 2026 Feb 20;6(2):e0005459. doi: 10.1371/journal.pgph.0005459 (PMC12923035; doi:10.1371/journal.pgph.0005459)
Supplement: S3 Table — (DOCX) [file pgph.0005459.s003.docx]

S3 Table. Characteristics and Evidence Synthesis of Included Studies Across Four Domains (n = 49)

| Reference | Country/Setting | Domain | Primary Population | Age | Gender | Pertinent Findings | Limitations | Recommendations |
| --- | --- | --- | --- | --- | --- | --- | --- | --- |
| Robinson et al. (2021) | Rwanda & Uganda | Mental Health | Congolese refugees | Adults | Mixed | Mental wellbeing was strongly shaped by social identity, dignity, and personal appearance. Programs that reinforced positive identity and community belonging helped reduce internalized stigma and encouraged help-seeking. | Overcrowded camp conditions, poverty, and limited specialist services restricted the sustainability of psychosocial initiatives. | Build culturally grounded, community-led mental health programs. |
| Kienzler (2024) | UK | Mental Health | Refugees/asylum seekers | Not specified | Not specified | Refugee-co-designed services were perceived as more respectful, responsive, and empowering than top-down models. Integration of social, legal, and mental health support was critical to effectiveness. | Immigration regulations and short-term project funding undermined long-term continuity and trust. | Remove policy barriers and institutionalize participatory design. |
| Paudyal et al. (2021) | UK | Mental Health | Syrian refugees | Adults | Mixed | Mental health services aligned with religious values and community structures were more acceptable and perceived as safer spaces for disclosure. | Limited interpreter availability and stigma within communities reduced access. | Integrate faith-sensitive approaches and community outreach. |
| Walther et al. (2021) | Germany | Mental Health | Refugees | Adults | Mixed | Social integration opportunities, language learning, and peer networks were key protective factors for psychological resilience. | Uncertainty of legal status and bureaucratic complexity worsened stress and hindered recovery. | Strengthen long-term social inclusion mechanisms. |
| Laham et al. (2020) | Lebanon | Mental Health | Syrian refugees & hosts | Adults | Mixed | Community mental health services that minimized stigma and were locally accessible increased willingness to seek care. | Transportation costs, fear of labeling, and economic hardship limited regular attendance. | Expand decentralized, community-based care. |
| Bridi et al. (2023) | Jordan & USA | Mental Health | Older refugees | Older adults | Mixed | Cultural beliefs and faith traditions shaped understanding of cognitive decline; faith-integrated counseling improved coping and acceptance. | Cultural stigma around dementia led to late presentation to services. | Engage religious leaders in mental health education. |
| Khan et al. (2022) | Canada | Mental Health | Refugee youth | Youth | Mixed | Stable mentoring relationships and youth-friendly spaces fostered resilience and a sense of safety among homeless refugee youth. | Housing instability and insecure immigration status undermined program consistency. | Combine psychosocial support with housing initiatives. |
| Ahmed et al. (2024) | Kenya | Mental Health | Somali refugees | Adults | Mixed | Trauma-informed, peer-supported approaches were culturally acceptable and helped normalize mental health discussions. | Extreme poverty and fragmented NGO programs limited continuity of care. | Invest in community peer networks. |
| Jensen et al. (2013) | Denmark | Mental Health | Refugee patients | Not reported | Not reported | General practitioners recognized that cultural competence training improved diagnostic accuracy and communication with refugee patients. | Providers lacked structured guidance for complex trauma care. | Integrate refugee-specific training in primary care. |
| Silver et al. (2023) | UK | Mental Health | Refugees & trainees | Adults | Mixed | Direct interaction with refugees transformed medical student attitudes and improved empathy toward migrant mental health needs. | One-off learning encounters limited sustained impact. | Embed long-term experiential learning in curricula. |
| Fabian et al. (2023) | USA | Mental Health | Refugee youth | Youth | Mixed | Culturally tailored digital interventions increased engagement and provided safe avenues for discussing emotional difficulties. | Digital exclusion and distrust of online platforms reduced uptake. | Adapt technology to local contexts and privacy concerns. |
| Callender et al. (2022) | USA | Mental Health | Muslim refugee women | Adults | Women | Prayer, spirituality, and mindfulness were central coping mechanisms and should be integrated into therapeutic models. | Fear of gossip, stigma, and confidentiality concerns reduced participation. | Offer women-only, faith-sensitive services. |
| Shannon et al. (2015) | USA | Mental Health | Mixed refugees | Adults | Mixed | Psychoeducation and peer facilitation helped participants reinterpret trauma experiences and reduce shame. | Deep mistrust of authorities limited engagement. | Expand peer-led stigma reduction programs. |
| Marshall & Barrett (2025) | Rwanda | Disability | Refugees with disabilities | Adults | Mixed | Tailored communication strategies enabled refugees with disabilities to understand and report gender-based violence risks. | Lack of accessible materials and assistive technologies limited reach. | Invest in disability-inclusive communication. |
| Mirza et al. (2013) | USA | Disability | Refugees with disabilities | Adults | Mixed | Mapping of service pathways revealed major unmet needs and the importance of culturally competent navigation assistance. | Transportation costs and inaccessible facilities were persistent barriers. | Develop community health navigation systems. |
| Kroening et al. (2016) | USA | Disability | Refugee children | Children | Mixed | Early developmental screening facilitated timely referrals and school integration for refugee children. | Lack of trained interpreters and culturally adapted tools hindered accuracy. | Standardize culturally adapted screening. |
| Tofani et al. (2025) | Italy | Disability | Refugees with disabilities | Not specified | Not reported | Coordinated rehabilitation services along migration routes improved continuity of care and reduced loss to follow-up. | Unsafe travel conditions and fragmented policies disrupted treatment. | Strengthen cross-border coordination. |
| Harris & Roberts (2003) | Multi-country | Disability | Disabled refugees | Adults | Mixed | Participatory research approaches empowered disabled refugees and improved program relevance. | Physical inaccessibility and institutional gatekeeping limited inclusion. | Ensure accessibility in research and services. |
| Avci & Sengul (2024) | Turkey | Disability | Syrian women | Adults | Women | Intersectional programs that addressed both gender and disability needs were more responsive and empowering. | Multiple layers of discrimination reduced participation. | Expand peer-support networks for women. |
| Mirza & Heinemann (2012) | USA | Disability | Refugees with disabilities | Adults | Mixed | Integrated case management reduced service fragmentation and improved care coordination. | Agencies operated in silos with poor communication. | Implement coordinated case management. |
| Bacakova (2025) | Germany | Disability | Ukrainian children | Children | Mixed | Inclusive classrooms enhanced social integration and educational outcomes. | Language barriers and limited special education resources. | Train teachers in inclusive methods. |
| Fayad et al. (2024) | Jordan | Disability | Caregivers | Children | Mixed | Mindfulness training strengthened caregiver coping and reduced burnout. | Program reached only small groups due to funding limits. | Scale accessible caregiver supports. |
| Serrano & Martin (2021) | Brazil | Disability | Disabled refugees | Not reported | Not reported | Legal frameworks recognized rights but practical access to services remained weak. | Large gap between policy intent and implementation. | Strengthen accountability mechanisms. |
| Scheer & Mondaca (2022) | Europe | Disability | Women with disabilities | Adults | Women | Leadership initiatives increased confidence and civic participation. | Ongoing social stigma and exclusion. | Support inclusive leadership training. |
| Kasper et al. (2021) | Multi-country | Women’s Health | Refugee women | Adults | Women | Maternal health programs using cultural mediators improved antenatal attendance and trust. | Language barriers and unfamiliarity with systems. | Institutionalize interpreter services. |
| Adıbelli & Şahan (2025) | Turkey | Women’s Health | Women & children | Adults/children | Women focused | Multi-sectoral collaborations improved access to social protection and health services. | Economic insecurity limited continuity. | Strengthen NGO-government coordination. |
| Griffin et al. (2022) | Australia | Women’s Health | Myanmar women | Adults | Women | Peer-led information sessions increased health literacy and system navigation. | Digital divide limited information access. | Expand face-to-face peer education. |
| Babatunde-Sowole (2020) | Australia | Women’s Health | Refugee women | Adults | Women | Preventive screening delivered in culturally sensitive settings increased participation. | Fear of authorities and past trauma. | Use female providers and community trust-builders. |
| Due et al. (2022) | Australia | Women’s Health | African women | Adults | Women | Integrated perinatal care models improved psychological wellbeing and continuity. | Cultural mismatch between staff and clients. | Build long-term provider relationships. |
| Woodgate et al. (2017) | Canada | Women’s Health | Families | Adults/children | Mixed | Community health workers were crucial bridges between refugees and primary care. | Complex systems discouraged access. | Employ patient navigators. |
| McMorrow et al. (2017) | Unspecified | Women’s Health | Congolese women | Adults | Women | Culturally responsive health promotion built trust and engagement. | Interpreter shortages. | Expand peer networks. |
| Baird et al. (2015) | Unspecified | Women’s Health | Sudanese women | Adults | Women | Co-leadership by refugee women increased program sustainability. | Institutional distrust limited early uptake. | Develop community leadership models. |
| Wu (2015) | USA | End-of-Life | Chinese caregivers | Adults | Mixed | Family-centered decision-making improved satisfaction and dignity at end of life. | Communication difficulties with providers. | Incorporate family rituals. |
| Bell (2018) | Australia | End-of-Life | Refugee patients | Adults | Not reported | Clinic environments designed with refugee input fostered trust and comfort. | Physical space and resource constraints. | Create culturally welcoming spaces. |
| Jansky (2019) | Germany | End-of-Life | Migrant patients | Adults | Mixed | Equity-oriented palliative models reduced perceived discrimination. | Providers lacked cultural training. | Implement ongoing staff education. |
| Najjar (2020) | Nepal | End-of-Life | Camp refugees | Adults | Mixed | Adapted low-resource palliative approaches preserved dignity. | Severe shortages of medicines and staff. | Mobilize community volunteers. |
| Abdelaal (2021) | Canada | End-of-Life | Refugee claimants | Adults | Not reported | Trust-based care relationships were essential for comfort and adherence. | Legal and policy barriers restricted access. | Advocate flexible service eligibility. |
| Doherty (2020) | Bangladesh | End-of-Life | Rohingya refugees | Adults | Mixed | Community linkages reduced suffering and unmet symptom needs. | Limited trained workforce. | Collaborate with local NGOs. |
| Molnar (2020) | Multi-setting | End-of-Life | Systems level | N/A | N/A | Integrating palliative care into primary health systems improved coordination and continuity. | Fragmented referral pathways. | Align palliative and PHC structures. |
| De Laat (2021) | Jordan & Rwanda | End-of-Life | Camp refugees | Adults | Mixed | NGO–health sector partnerships enhanced service coordination. | Funding instability. | Institutionalize partnerships. |
| de Voogd (2021) | Netherlands | End-of-Life | Migrant caregivers | Adults | Mixed | Dignity-focused communication improved family experiences. | Cultural misunderstandings. | Train staff in dignity-centered care. |
| Shabnam et al. (2024) | Denmark | End-of-Life | Migrant patients | Adults | Mixed | Interpreter use improved shared decision-making. | Interpreter shortages. | Expand language services. |
| Ashrafizadeh (2023) | EMR region | End-of-Life | Migrants/refugees | Adults | Mixed | Regional collaboration models strengthened culturally responsive care. | Limited resources and political will. | Develop cross-border networks. |
| Cummins (2023) | Canada | End-of-Life | Muslim migrants | Adults | Mixed | Alignment with religious values enhanced dignity and acceptance of care. | Tension between biomedical and faith models. | Engage faith leaders. |
| Hudson et al. (2023) | UK | End-of-Life | Homeless migrants | Adults | Mixed | Mobile outreach improved access for highly marginalized patients. | Unstable housing undermined continuity. | Deploy mobile palliative teams. |
| Zhang et al. (2023) | USA | End-of-Life | Immigrant/refugee clients | Adults | Mixed | Linking HIV and palliative services increased uptake. | Persistent stigma and language barriers. | Use peer navigators. |
| De Laat (2024) | Rwanda | End-of-Life | Camp refugees | Adults | Mixed | Locally adapted models were highly acceptable to communities. | Staff and supply shortages. | Train local health workers. |
| Leng et al. (2024) | Uganda | End-of-Life | Refugee settlements | Adults | Mixed | Participatory appraisals improved integration of services. | Fragmented governance. | Conduct joint training. |
| Gupta et al. (2025) | Urban hospitals | End-of-Life | Refugee patients | Adults | Mixed | Transitional supports reduced unnecessary hospital deaths and improved planning. | Lack of home-based care options. | Expand discharge planning. |
