# Supplementary material for: Community health assets and refugee wellbeing: Qualitative evidence across mental health, disability inclusion, end-of-life care, and women’s health – A global scoping review
Source: PLOS Glob Public Health. 2026 Feb 20;6(2):e0005459. doi: 10.1371/journal.pgph.0005459 (PMC12923035; doi:10.1371/journal.pgph.0005459)
Supplement: S4 Table — (DOCX) [file pgph.0005459.s004.docx]

**S4 Table.** Critical Appraisal of Included Qualitative Studies Using the CASP

| Study | Aim Clearly Stated | Qualitative Method Appropriate | Research Design Justified | Recruitment Strategy Appropriate | Data Collection in Line with Aims | Researcher-Participant Reflexivity | Ethical Issues Considered | Data Analysis Sufficiently Rigorous | Clear Statement of Findings | Research Value |
| --- | --- | --- | --- | --- | --- | --- | --- | --- | --- | --- |
| Robinson et al. (2022) | Yes | Yes | Yes | No | Yes | Yes | No | Partial | Yes | Moderate |
| Kienzler (2024) | Yes | Yes | Yes | Partial | Yes | Partial | Partial | Yes | Yes | Moderate |
| Paudyal et al. (2021) | Yes | Yes | Yes | Yes | Yes | Partial | Yes | Yes | Yes | Moderate |
| Walther et al. (2021) | Yes | Yes | Yes | No | Yes | Partial | Partial | No | Yes | Moderate |
| Al Laham et al. (2020) | Yes | Yes | Yes | Partial | Yes | Yes | Partial | No | Yes | Moderate |
| Bridi et al. (2023) | Yes | Yes | Yes | Yes | Yes | Partial | Yes | Yes | Yes | Moderate |
| Khan et al. (2022) | Yes | Yes | Yes | Yes | Yes | No | No | No | Yes | Low |
| Ahmed et al. (2024) | Yes | Yes | Yes | No | Yes | Partial | Partial | Yes | Yes | Low |
| Jensen et al. (2013) | Yes | Yes | Yes | Partial | Yes | Partial | No | No | Yes | High |
| Silver et al. (2023) | Yes | Yes | Yes | No | Yes | Partial | Yes | No | Yes | Moderate |
| Fabian et al. (2025) | Yes | Yes | Yes | Yes | Yes | Partial | No | No | Yes | Low |
| Callender et al. (2022) | Yes | Yes | Yes | No | Yes | No | No | Yes | Yes | High |
| Shannon et al. (2015) | Yes | Yes | Yes | Partial | Yes | No | Yes | Yes | Yes | Low |
| Marshall & Barrett (2025) | Yes | Yes | Yes | Yes | Yes | Partial | No | Partial | Yes | Moderate |
| Mirza et al. (2014) | Yes | Yes | Yes | Yes | Yes | No | Yes | No | Yes | High |
| Kroening et al. (2016) | Yes | Yes | Yes | Yes | Yes | Partial | Partial | Yes | Yes | Low |
| Tofani et al. (2023) | Yes | Yes | Yes | No | Yes | No | Partial | Yes | Yes | Low |
| Harris & Roberts (2003) | Yes | Yes | Yes | Partial | Yes | Yes | Yes | No | Yes | Low |
| Avci & Sengul (2024) | Yes | Yes | Yes | Yes | Yes | Yes | Partial | Partial | Yes | Low |
| Mirza & Heinemann (2012) | Yes | Yes | Yes | Yes | Yes | Yes | Yes | Partial | Yes | High |
| Bacakova (2025) | Yes | Yes | Yes | Yes | Yes | Yes | Yes | Partial | Yes | High |
| Fayad et al. (2024) | Yes | Yes | Yes | Yes | Yes | Partial | No | Partial | Yes | Low |
| Serrano & Martin (2021) | Yes | Yes | Yes | Partial | Yes | Yes | Yes | No | Yes | Low |
| Scheer & Mondaca (2022) | Yes | Yes | Yes | Yes | Yes | No | No | Yes | Yes | Moderate |
| Kasper et al. (2022) | Yes | Yes | Yes | No | Yes | Yes | Partial | No | Yes | Moderate |
| Adibelli & Sahan (2025) | Yes | Yes | Yes | No | Yes | Yes | Partial | No | Yes | High |
| Griffin et al. (2022) | Yes | Yes | Yes | Partial | Yes | Partial | Yes | Partial | Yes | High |
| Babatunde-Sowole et al. (2020) | Yes | Yes | Yes | Partial | Yes | No | Yes | Partial | Yes | High |
| Due et al. (2022) | Yes | Yes | Yes | Yes | Yes | Yes | Partial | No | Yes | Moderate |
| Woodgate et al. (2017) | Yes | Yes | Yes | Yes | Yes | No | Yes | No | Yes | Low |
| McMorrow & Saksena (2017) | Yes | Yes | Yes | Yes | Yes | Yes | No | Partial | Yes | Moderate |
| Baird et al. (2015) | Yes | Yes | Yes | No | Yes | No | Yes | Partial | Yes | Low |
| Wu (2015) | Yes | Yes | Yes | Yes | Yes | Yes | No | Partial | Yes | Low |
| Bell (2018) | Yes | Yes | Yes | Partial | Yes | No | No | Yes | Yes | High |
| Jansky et al. (2019) | Yes | Yes | Yes | No | Yes | No | Partial | No | Yes | Low |
| Najjar & Hauck (2020) | Yes | Yes | Yes | Yes | Yes | Partial | Yes | No | Yes | Low |
| Abdelaal et al. (2021) | Yes | Yes | Yes | No | Yes | Yes | No | Yes | Yes | Moderate |
| Doherty et al. (2020) | Yes | Yes | Yes | Partial | Yes | Yes | No | No | Yes | Low |
| Molnar & Isaac (2020) | Yes | Yes | Yes | Partial | Yes | No | No | No | Yes | High |
| de Laat et al. (2021) | Yes | Yes | Yes | No | Yes | Yes | Yes | Partial | Yes | Low |
| de Voogd et al. (2021) | Yes | Yes | Yes | Yes | Yes | Partial | Partial | Yes | Yes | Low |
| Shabnam et al. (2024) | Yes | Yes | Yes | No | Yes | Partial | Partial | No | Yes | High |
| Ashrafizadeh & Rassouli (2023) | Yes | Yes | Yes | No | Yes | Partial | No | Yes | Yes | Moderate |
| Cummins & Dossa (2020) | Yes | Yes | Yes | No | Yes | Yes | Partial | No | Yes | High |
| Hudson et al. (2023) | Yes | Yes | Yes | No | Yes | Partial | Partial | Yes | Yes | Low |
| Zhang et al. (2023) | Yes | Yes | Yes | Yes | Yes | No | Yes | No | Yes | High |
| de Laat et al. (2024) | Yes | Yes | Yes | Partial | Yes | Yes | No | No | Yes | Low |
| Leng et al. (2024) | Yes | Yes | Yes | Yes | Yes | Yes | Partial | Yes | Yes | Low |
| Gupta et al. (2025) | Yes | Yes | Yes | No | Yes | Yes | Partial | No | Yes | High |
