# Supplementary material for: Community health assets and refugee wellbeing: Qualitative evidence across mental health, disability inclusion, end-of-life care, and women’s health – A global scoping review
Source: PLOS Glob Public Health. 2026 Feb 20;6(2):e0005459. doi: 10.1371/journal.pgph.0005459 (PMC12923035; doi:10.1371/journal.pgph.0005459)
Supplement: Attachment — Submitted filename: Response to Reviewers.docx [file pgph.0005459.s007.docx]

## COVER LETTER

Subject: Revised Manuscript Submission – PGPH-D-25-03306

Dear Dr. Diab,

Thank you for the opportunity to revise and resubmit our manuscript, “Community Health Assets and Refugee Wellbeing: Qualitative Evidence across Mental Health, Disability Inclusion, End-of-Life Care, and Women’s Health – A Global Scoping Review” (Manuscript ID: PGPH-D-25-03306). We are grateful for the careful and constructive feedback provided by the reviewers and the editorial team.

We have revised the manuscript extensively in response to these comments. In particular, we have strengthened the presentation of study population characteristics, clarified the scope regarding child health, and expanded our analysis of differences between high-income and low- and middle-income country contexts. We have also clarified key conceptual distinctions among refugees, asylum seekers, and internally displaced persons, elaborated our methods for handling studies spanning multiple domains, and corrected inconsistencies in figures and tables. The Limitations section has been expanded to address geographic and income-level imbalances in the available evidence.

All journal requirements have been fully addressed. The Financial Disclosure and Competing Interests statements have been updated as requested. A detailed point-by-point response to reviewers, together with a tracked-changes version of the revised manuscript, accompanies this submission.

We appreciate the guidance we received through this review process and believe that the manuscript has been substantially strengthened as a result. We hope the revised version will now be suitable for publication in PLOS Global Public Health.

Sincerely,
Dr. Sileshi Demelash Sasie
Ethiopian Public Health Institute
Addis Ababa, Ethiopia

# **RESPONSE TO JOURNAL REQUIREMENTS**

We thank the editorial office for the clear and helpful instructions. Each requirement has been addressed as outlined below.

**1. Financial Disclosure Statement**: The online Financial Disclosure statement has been amended as requested. It now reads: The authors received no specific funding for this work.

**2. Competing Interests Statement**: The Competing Interests statement has been updated to read: The authors have declared that no competing interests exist.

**3. Figure Files**: All figures have been prepared and uploaded as separate files in the required .tif or .eps formats. Any figures previously embedded within the manuscript have been removed, while figure captions have been retained in the manuscript text. All files are within the journal size limit.

**4. Supplementary Material File Type**: The file previously uploaded as “Supplementary material.docx” under the “Other” category has been re-uploaded as “Supporting Information” to ensure full accessibility to reviewers. Relevant references within the manuscript have been updated accordingly.

**5. Legends for Supporting Information**: A complete list of legends for all Supporting Information files has been added to the manuscript immediately following the reference list, as requested.

We appreciate the opportunity to address these requirements and confirm that all requested changes have now been completed.

# RESPONSE TO REVIEWER COMMENTS

## Reviewer 2

**Comment 1: Study population characteristics and demographics**

Please provide clearer demographic information on the populations included in the reviewed studies (e.g., age, gender, geographic distribution where available).

**Response**: We thank the reviewer for this important suggestion. In response, we have strengthened demographic reporting across the manuscript. A consolidated summary table (S3 Table) has been added to the Results to present, for each study, the country or setting, primary population, and available age and gender information. We have also added a new subsection titled “Study Population Characteristics” to synthesize these data narratively. In addition, the Methods section now more clearly describes how demographic variables were extracted and coded.

**Revision**: Demographic variables were recorded exactly as reported in the primary studies and categorized as age group, gender composition, and geographic setting. Where information was not available, it was coded as “not reported” rather than inferred in order to maintain transparency (Methods section, page 13, lines 266–271).

**Comment 2: Scope and treatment of child health**

Please justify more explicitly why child health was excluded from the scope, or revise the review to incorporate child health.

**Response**: We appreciate this thoughtful comment. The review was deliberately structured around four defined domains mental health, disability inclusion, women’s health, and end-of-life and palliative care each representing distinct areas of qualitative evidence. Importantly, no age restriction was applied within these domains, and studies involving children and adolescents were included whenever they addressed one of the four thematic areas. Broader topics in general child health were not excluded due to lack of relevance, but because they constitute a large and heterogeneous field that lies outside the specific analytic focus of this review.

**Revision**: Section 2.3 (Eligibility Criteria) has been revised to clarify those studies including children were eligible when relevant to the focal domains, while broader pediatric health topics were outside the defined scope (Methods section, page 8, lines 157–158).

**Comment 3: High-income vs. low- and middle-income country contexts**

Please strengthen your analysis by more explicitly distinguishing between these contexts and reflecting on how structural constraints, service provision, and community health assets differ across income settings, including the relatively limited representation of LMICs.

**Response**: We thank the reviewer for this valuable recommendation and fully agree that clearer differentiation between high-income country (HIC) and low- and middle-income country (LMIC) contexts strengthens the interpretation of our findings.

To address this point, we have made the following revisions:

1. **New Results subsection**: A dedicated subsection titled “Differences Across Income Settings” has been added to the Results. This section systematically compares HIC and LMIC contexts, describing differences in structural constraints, service delivery models, and the types of community health assets reported across settings (Results section, pages 40–44, lines 581–601).
2. **Expanded Discussion**: The Discussion has been strengthened with new analytic text contrasting reliance on formal health-system assets in HIC settings with community- and NGO-based platforms in LMIC contexts. This new material interprets how these contextual differences influence feasibility, acceptability, and sustainability of interventions, emphasizes the importance of cultural adaptation and participatory design, and cautions against uncritical transfer of HIC-based models to LMIC environments (Discussion section, pages 47–48, lines 660–683).
3. **Enhanced Limitations**: The Limitations section now explicitly acknowledges the predominance of studies from HIC settings and the relatively limited qualitative evidence from LMICs, where most displaced populations reside (Strengths and Limitations section, lines 720–724).

## **Reviewer 1**

**Comment 1: Clarifying distinctions between refugees, asylum seekers, and other forcibly displaced populations**

**Response**: We appreciate the reviewer’s emphasis on conceptual clarity. In the revised manuscript, we have strengthened the definitions and distinctions between refugees, asylum seekers, and internally displaced persons (IDPs), aligning them with established international legal and policy frameworks. These distinctions are now consistently articulated throughout the manuscript to avoid conflation of categories.

**Revisions**

- A dedicated paragraph has been added to the Background section defining these groups using internationally recognized terminology (Background section, page 3, lines 56–68).
- The Population component of the PCC framework and the Eligibility Criteria have been revised to explicitly specify these categories (Methods section, page 7, line 517).

**Comment 2: Explaining how studies spanning multiple thematic domains were classified and analyzed**

**Response**: We thank the reviewer for highlighting this methodological point. The Methods section has been revised to provide a clearer explanation of how studies addressing more than one domain were handled.

**Revisions**

- Additional text has been added to clarify that such studies were coded under all relevant domains during data extraction (Methods section, page 13, lines 259–262).
- Section 2.7 (Data Synthesis) now explicitly explains that procedures were used to incorporate information from multi-domain studies appropriately while avoiding duplication in aggregate analyses (Methods section, page 13, lines 263–267).

**Comment 3: Correcting inconsistencies and errors in Figure 1 and tables**

**Response**: We thank the reviewer for drawing attention to this issue. All figures and tables have been carefully reviewed and corrected to ensure internal consistency and alignment with the final dataset.

**Revisions**

- Figure 1 has been comprehensively revised to correct numerical inconsistencies and ensure full alignment with the study selection process described in the text.
- All tables were reviewed and updated for formatting consistency and accuracy.
- Legends and abbreviations have been standardized across the manuscript.

**Comment 4: Explicitly reflecting inclusion of children in the population criteria**

**Response**: We appreciate this helpful suggestion.

**Revisions**

- The Eligibility Criteria section now clearly states that studies involving children and adolescents were eligible when relevant (Methods section, lines 166–170).
- The Methods section explicitly notes that no age restrictions were applied (Methods section, line 157).
- The Data Extraction section clarifies that age group information was extracted whenever reported (Methods section, line 232).

**Comment 5: Correcting typographical errors and inconsistencies**

**Response**: The manuscript has undergone a thorough language and formatting review.

**Revisions**

- Typographical and grammatical errors have been corrected throughout.
- All abbreviations are now defined at first use and applied consistently.
- Terminology has been harmonized across all sections.

**Comment 6: Ensuring supplementary materials are properly uploaded and accessible**

**Response and Revisions**

- All supplementary files have been reviewed for completeness and accuracy.
- In-text references have been updated to correspond precisely with file names.
- All materials have been correctly uploaded and confirmed as accessible.

**Comment 7: Expanding the limitations section to address geographic and income-level imbalances**

**Response**: We appreciate this important recommendation.

**Revision**: New text has been added to the Limitations section explicitly discussing the predominance of studies from high-income settings and the relative scarcity of evidence from low- and middle-income countries (Strengths and Limitations section, lines 720–724).

We thank both reviewers and the editorial team for their constructive feedback, which has significantly strengthened the manuscript.
