# Supplementary material for: Community health assets and refugee wellbeing: Qualitative evidence across mental health, disability inclusion, end-of-life care, and women’s health – A global scoping review
Source: PLOS Glob Public Health. 2026 Feb 20;6(2):e0005459. doi: 10.1371/journal.pgph.0005459 (PMC12923035; doi:10.1371/journal.pgph.0005459)
Supplement: Attachment — Submitted filename: Point by point response.docx [file pgph.0005459.s008.docx]

Dear Dr. Diab,

Thank you for your decision letter and for the continued constructive engagement with our manuscript. We appreciate the opportunity to submit a revised version following the re-review process.

We have carefully addressed all remaining comments from the reviewers, further strengthening population clarity, conceptual framing, and analytical transparency, and resolving all outstanding technical and presentation issues. The detailed point-by-point responses to each reviewer comment are provided below.

We trust that these revisions satisfactorily address the points raised and that the manuscript is now well positioned for final consideration in *PLOS Global Public Health*.

Kind regards,

Sileshi Demelash Sasie

(on behalf of all authors)

**Reviewer Comment 1: Population clarity and demographics**

**Comment:** Please provide basic demographic characteristics of the study populations where available. In addition, clearly distinguish between refugees, asylum seekers, and forcibly displaced people throughout the manuscript, as these terms are currently used interchangeably. Several included studies involve children, so please also reflect this explicitly in your population inclusion criteria (e.g., "refugees of all ages").

**Response:**
We thank the reviewer for this important and constructive comment. We have substantially strengthened population clarity and demographic transparency in the revised manuscript. First, we now explicitly distinguish between refugees, asylum seekers, and internally displaced persons (IDPs) in the Background section, using internationally recognised legal definitions. These terms are applied consistently throughout the manuscript to avoid conceptual conflation and to reflect their distinct legal and health system implications **(Background, lines 60–69).**

Second, we revised the population inclusion criteria to explicitly include refugees, asylum seekers, and internally displaced persons of all ages. Studies involving children and adolescents were retained when they addressed issues directly relevant to one or more of the four focal domains (for example, refugee youth mental health or disability-related services for children). This clarification is now clearly stated in the Eligibility Criteria section of the Methods **(lines 159–172).**

Third, we expanded the data extraction framework to systematically capture available demographic characteristics across all included studies. Specifically, we extracted and reported primary population category (refugees, asylum seekers, IDPs, or mixed), age group (children, adolescents, adults, and older adults), gender composition, and geographic setting. These variables are described in detail in the Methods section and summarised in the Results and supplementary tables **(Methods, lines 232–236; Results, lines 337–338)**

**Reviewer Comment 2: Scope and conceptual framing**

**Comment:** One reviewer noted the absence of child health as a domain. Please either justify its exclusion or explain how it could be incorporated. Relatedly, we ask that you more explicitly reflect on the challenges of synthesizing findings across high- and low-income settings. Given the relatively small proportion of LMIC studies and the very different barriers across contexts, this should be discussed more critically, including in the Limitations section.

**Response:**
We appreciate this important conceptual observation and have addressed it through clarification of scope in the revised manuscript. With regard to child health, we have explicitly justified its exclusion as a standalone domain. Although children and adolescents are included across all four focal areas, the review is structured around domains defined by specific health system functions (mental health, disability inclusion, women’s health, and end-of-life and palliative care). The objective was not to provide a comprehensive pediatric health review, but rather to synthesise implementation-relevant qualitative evidence within these analytically coherent domains. Broader child health topics, such as general morbidity, nutrition, or immunization, were therefore considered outside the conceptual scope of this review. This rationale is articulated in the Eligibility Criteria **(Methods section, lines 159–172).**

In response to the second point, we have substantially strengthened the manuscript’s treatment of contextual heterogeneity across income settings. A new dedicated subsection now presents a structured synthesis of how intervention models, delivery platforms, and enabling assets differ between high-income resettlement contexts and low- and middle-income or humanitarian settings. These revisions are reflected in the Results section **(lines 588–600).**

Furthermore, the Limitations section now explicitly acknowledges the predominance of high-income studies, the underrepresentation of LMIC evidence, and the implications this has for generalizability, transferability, and interpretive caution. We also critically reflect on how differences in health system capacity, policy environments, and resource constraints complicate cross-context synthesis and limit the direct portability of certain intervention models **(Limitations, lines 721–725).**

**Reviewer Comment 3: Analytical transparency across domains**

**Comment**: Please clarify how studies spanning multiple domains (e.g., women’s health and mental health) were classified and analyzed. For example, some studies categorized under Women’s Health clearly intersect with Mental Health. A brief explanation of your analytical approach to overlap and intersection would improve clarity.

**Response:**
We fully agree with this point and have clarified our analytical approach to overlapping domains in the Data Synthesis section. Studies addressing more than one thematic domain were coded under all applicable domains during data extraction; however, for cross-domain analyses and aggregate summaries, each study was treated as a single analytic unit and counted only once. This methodological clarification enhances analytical transparency and avoids double counting of intersecting evidence **(Methods section, lines 263–267).**

**Reviewer Comment 4.1: PRISMA Figure 1**

**Comment:** Please include grey literature and Google Scholar records in the main identification box. Correct the numerical discrepancy in the final boxes (125 − 77 = 48, not 49).

**Response:**
We have revised PRISMA Figure 1 to explicitly include grey literature and Google Scholar records within the main identification box, in accordance with PRISMA-ScR reporting standards. We also corrected the numerical discrepancy in the final screening boxes, and the flow diagram now accurately reflects the study selection process, with all counts internally consistent.

**Reviewer Comment 4.2: End-of-life section**

**Comment:** Please verify the reported number of EOL/palliative care studies (there appears to be an inconsistency). In Table 5, ensure “et al.” is consistently applied in citations, as in earlier tables.

**Response:**
We re-verified the number of end-of-life and palliative care studies and corrected the reported totals to ensure consistency between the Results narrative, domain-level synthesis, and corresponding tables. We also reviewed Table 5 and standardized citation formatting so that “et al.” is applied consistently across all entries, in line with the formatting used in earlier tables.

**Reviewer Comment 4.3: Abbreviations and consistency**

**Comment:** Define NHS, WHO, UNHCR, NGOs, PTSD, SDGs at first use and ensure they appear in the Abbreviations list. Standardize usage of "gender-based violence (GBV)" throughout. Correct minor typographical issues (including a duplicated comma in the Background).

**Response:** We ensured that NHS, WHO, UNHCR, NGOs, PTSD, and SDGs are now defined at first mention in the main text and included in the Abbreviations list. We also standardised terminology for gender-based violence by using the format “gender-based violence (GBV)” consistently throughout the manuscript. All identified typographical issues, including the duplicated comma in the Background section, were corrected following a full proofreading of the revised version.

**Reviewer Comment 4.4: Supplementary materials**

**Comment:** The supplementary tables and figures referenced in the manuscript were not accessible. Please ensure all supplementary files are uploaded and clearly labeled.

**Response:** All supplementary tables and figures referenced in the manuscript have now been uploaded as separate files and clearly labelled using the journal’s required format (e.g., S1 Table, S2 Table, S3 Table). Cross-references within the main text were reviewed and corrected to ensure accurate alignment with the uploaded supplementary materials.
