# Supplementary material for: Community health assets and refugee wellbeing: Qualitative evidence across mental health, disability inclusion, end-of-life care, and women’s health – A global scoping review
Source: PLOS Glob Public Health. 2026 Feb 20;6(2):e0005459. doi: 10.1371/journal.pgph.0005459 (PMC12923035; doi:10.1371/journal.pgph.0005459)
Supplement: Peer review history

# Peer review: Decision Letter 0

**DOI:** 10.1371/journal.pgph.0005459.r001

**Authors:** Jasmin Lilian Diab

9 Jan 2026

PGPH-D-25-03306

Community Health Assets and Refugee Wellbeing: Qualitative Evidence across Mental Health, Disability Inclusion, End-of-Life Care, and Women’s Health – A Global Scoping Review

PLOS Global Public Health

Dear Authors,

Thank you for submitting your manuscript, 'Community Health Assets and Refugee Wellbeing: Qualitative Evidence across Mental Health, Disability Inclusion, End-of-Life Care, and Women’s Health – A Global Scoping Review' to PLOS Global Public Health.

After careful consideration of the two external peer reviews, I am writing to inform you that the editorial decision is Major Revision.

Both reviewers recognize the value, relevance, and overall rigor of your study. Reviewer 1 considers the manuscript technically sound and well conducted, and Reviewer 2 highlights the importance of the topic and the robustness of the scoping review methodology. However, Reviewer 2 has raised several substantive concerns that require further clarification and revision before the manuscript can be considered for publication.

In particular, you are asked to address the following major issues:

1. Study population characteristics and demographics: Please provide clearer demographic information on the populations included in the reviewed studies (e.g., age, gender, geographic distribution where available). This is critical for contextualizing the findings and understanding whose experiences are being captured.

2. Scope and treatment of child health: Reviewer 2 notes that child health is an important and currently underdeveloped area within your review. You should either:

* justify more explicitly why child health was excluded from the scope, or

* revise the review to incorporate child health and explain how it analytically fits within (or alongside) your four domains.

3. High-income vs. low- and middle-income country contexts: While the global scope of the review is appreciated, concerns were raised about how findings from high-income countries and low-/middle-income countries are synthesized. Please strengthen your analysis by:

* more explicitly distinguishing between these contexts, and

* reflecting on how structural constraints, service provision, and community health assets differ across income settings, including the relatively limited representation of LMICs.

In addition to these major points, you should also address the minor but important revisions raised by Reviewer 1, including but not limited to:

* Clarifying distinctions between refugees, asylum seekers, and other forcibly displaced populations.

* Explaining how studies spanning multiple thematic domains were classified and analyzed.

* Correcting inconsistencies and errors in Figure 1 and tables.

* Explicitly reflecting the inclusion of children in the population criteria, if applicable.

* Correcting typographical errors, missing abbreviations, and inconsistencies in terminology.

* Ensuring all supplementary materials referenced in the manuscript are properly uploaded and accessible.

* Expanding the limitations section to address geographic and income-level imbalances in the literature.

We believe that, with these revisions, your manuscript has strong potential to make a valuable contribution to the field. We look forward to receiving your revised submission and reviewing it further.

Please submit your revised manuscript by February 28. If you will need more time than this to complete your revisions, please reply to this message or contact the journal office at globalpubhealth@plos.org. When you're ready to submit your revision, log on to https://www.editorialmanager.com/pgph/ and select the 'Submissions Needing Revision' folder to locate your manuscript file.

Please include the following items when submitting your revised manuscript:

- A letter that responds to each point raised by the editor and reviewer(s). You should upload this letter as a separate file labeled 'Response to Reviewers'.
- A marked-up copy of your manuscript that highlights changes made to the original version. You should upload this as a separate file labeled 'Revised Manuscript with Track Changes'.
- An unmarked version of your revised paper without tracked changes. You should upload this as a separate file labeled 'Manuscript'.

We look forward to receiving your revised manuscript.

Peace,

Jasmin Lilian Diab, Ph.D

Guest Editor

PLOS Global Public Health

Journal Requirements

1. Please amend your online Financial Disclosure statement. If you did not receive any funding for this study, please simply state: “The authors received no specific funding for this work.”

2. Please update your online Competing Interests statement. If you have no competing interests to declare, please state: “The authors have declared that no competing interests exist.”

3. Please provide separate main figure files in .tif or .eps format only and remove any figures embedded in your manuscript file. Please also ensure that all files are under our size limit of 10MB. Please leave the figure captions in the manuscript.

For more information about how to convert your figure files please see our guidelines: https://journals.plos.org/globalpublichealth/s/figures

4. “Supplementary material.docx” is currently uploaded as an 'Other' file type, which is not viewable by reviewers. Please ensure that all files meant for review are uploaded as 'Supporting Information' and include a legend in the manuscript.

5. We have noticed that you have uploaded Supporting Information files, but you have not included a list of legends. Please add a full list of legends for your Supporting Information files before or after the references list.

# Author comment: Author response to Decision Letter 1

**DOI:** 10.1371/journal.pgph.0005459.r002

21 Jan 2026

Attachment Submitted filename: Response to Reviewers.docx

# Editor report: Decision Letter 1

**DOI:** 10.1371/journal.pgph.0005459.r003

**Authors:** Jasmin Lilian Diab

1 Feb 2026

PGPH-D-25-03306R1

Community Health Assets and Refugee Wellbeing: Qualitative Evidence across Mental Health, Disability Inclusion, End-of-Life Care, and Women’s Health – A Global Scoping Review

PLOS Global Public Health

Dear Authors,

Thank you for your thoughtful and timely submission. Both reviewers found the manuscript relevant and important, particularly appreciating its global scope, inclusion of low-income contexts, and overall methodological rigor. On this basis, we are inviting **minor revisions** prior to moving forward.

To strengthen the paper, please address the following points:

**1. Population clarity and demographics**

Please provide basic demographic characteristics of the study populations where available. In addition, clearly distinguish between *refugees*, *asylum seekers*, and *forcibly displaced people* throughout the manuscript, as these terms are currently used interchangeably. Several included studies involve children, so please also reflect this explicitly in your population inclusion criteria (e.g., "refugees of all ages").

**2. Scope and conceptual framing**

One reviewer noted the absence of child health as a domain. Please either justify its exclusion or explain how it could be incorporated.

Relatedly, we ask that you more explicitly reflect on the challenges of synthesizing findings across high- and low-income settings. Given the relatively small proportion of LMIC studies and the very different barriers across contexts, this should be discussed more critically, including in the Limitations section.

**3. Analytical transparency across domains**

Please clarify how studies spanning multiple domains (e.g., women’s health and mental health) were classified and analyzed. For example, some studies categorized under Women’s Health clearly intersect with Mental Health. A brief explanation of your analytical approach to overlap and intersection would improve clarity.

**4. Technical and presentation issues**

* PRISMA Figure 1:

* Please include grey literature and Google Scholar records in the main identification box.

* Correct the numerical discrepancy in the final boxes (125 − 77 = 48, not 49).

* End-of-life section:

* Please verify the reported number of EOL/palliative care studies (there appears to be an inconsistency).

* In Table 5, ensure “et al.” is consistently applied in citations, as in earlier tables.

* Abbreviations and consistency:

* Define NHS, WHO, UNHCR, NGOs, PTSD, SDGs at first use and ensure they appear in the Abbreviations list.

* Standardize usage of "gender-based violence (GBV)" throughout.

* Correct minor typographical issues (including a duplicated comma in the Background).

* Supplementary materials:

* The supplementary tables and figures referenced in the manuscript were not accessible. Please ensure all supplementary files are uploaded and clearly labeled.

Once these points are addressed, the manuscript will be much strengthened and well positioned for publication.

We look forward to receiving your revised version.

Peace,

Jasmin Lilian Diab, PhD

Guest Editor

PLOS Global Public Health

Journal Requirements:

If the reviewer comments include a recommendation to cite specific previously published works, please review and evaluate these publications to determine whether they are relevant and should be cited. There is no requirement to cite these works unless the editor has indicated otherwise.

Please review your reference list to ensure that it is complete and correct. If you have cited papers that have been retracted, please include the rationale for doing so in the manuscript text, or remove these references and replace them with relevant current references. Any changes to the reference list should be mentioned in the rebuttal letter that accompanies your revised manuscript. If you need to cite a retracted article, indicate the article’s retracted status in the References list and also include a citation and full reference for the retraction notice.

# Author comment: Author response to Decision Letter 2

**DOI:** 10.1371/journal.pgph.0005459.r004

4 Feb 2026

Attachment Submitted filename: Point by point response.docx

# Editor report: Decision Letter 2

**DOI:** 10.1371/journal.pgph.0005459.r005

**Authors:** Jasmin Lilian Diab

6 Feb 2026

Community Health Assets and Refugee Wellbeing: Qualitative Evidence across Mental Health, Disability Inclusion, End-of-Life Care, and Women’s Health – A Global Scoping Review

PGPH-D-25-03306R2

We are pleased to inform you that your manuscript 'Community Health Assets and Refugee Wellbeing: Qualitative Evidence across Mental Health, Disability Inclusion, End-of-Life Care, and Women’s Health – A Global Scoping Review' has been provisionally accepted for publication in PLOS Global Public Health.

Before your manuscript can be formally accepted you will need to complete some formatting changes, which you will receive in a follow up email. A member of our team will be in touch with a set of requests.

Please note that your manuscript will not be scheduled for publication until you have made the required changes, so a swift response is appreciated.

IMPORTANT: The editorial review process is now complete. PLOS will only permit corrections to spelling, formatting or significant scientific errors from this point onwards. Requests for major changes, or any which affect the scientific understanding of your work, will cause delays to the publication date of your manuscript.

If your institution or institutions have a press office, please notify them about your upcoming paper to help maximize its impact. If they'll be preparing press materials, please inform our press team as soon as possible -- no later than 48 hours after receiving the formal acceptance. Your manuscript will remain under strict press embargo until 2 pm Eastern Time on the date of publication. For more information, please contact globalpubhealth@plos.org.

Thank you again for supporting Open Access publishing; we are looking forward to publishing your work in PLOS Global Public Health.

Peace,

Jasmin Lilian Diab, Ph.D

Guest Editor

PLOS Global Public Health
